# Ubiquitin-like modifier-activating enzyme 1 interacts with Zika virus NS5 and promotes viral replication in the infected cell

**DOI:** 10.1099/jgv.0.002063

**Published:** 2025-01-08

**Authors:** Imanol Rodrigo, Laura Albentosa-González, María José Romero de Ávila, Maria Rosaria Bassi, Raquel Navarro Sempere, Pilar Clemente-Casares, Armando Arias

**Affiliations:** 1 Unidad de Medicina Molecular, Instituto de Biomedicina de UCLM (IB-UCLM), Universidad de Castilla-La Mancha (UCLM), Albacete, Spain; 2 Unidad de Biomedicina UCLM-CSIC, Albacete, Spain; 3 Facultad de Farmacia, UCLM, Albacete, Spain; 4 Centre for Translational Medicine and Parasitology at Department of Immunology and Microbiology (ISIM), Faculty of Health and Medical Sciences, University of Copenhagen, Copenhagen, Denmark; 5 Departamento de Biología Molecular, Investigación y Desarrollo de Ensayos Agroalimentarios SL (IDEAGRO an Alltech Company), 30564 Lorquí, Spain; 6 Escuela Técnica Superior de Ingenieros Agrónomos y de Montes y Biotecnología (ETSIAMB), UCLM, Albacete, Spain

**Keywords:** host–virus interactions, orthoflavivirus, RNA-dependent RNA polymerase, RNA virus replication, Usutu virus, ubiquitin-proteasome system

## Abstract

Translation errors, impaired folding or environmental stressors (e.g. infection) can all lead to an increase in the presence of misfolded proteins. These activate cellular responses to their removal, including intracellular protein degradation activities. Protein ubiquitylation is involved in two major degradation pathways, the ubiquitin-proteasome system and selective autophagy. In humans, the ubiquitin-like modifier-activating enzyme 1 (UBA1) is the primary E1 enzyme in the ubiquitin conjugation cascade. Viruses have evolved to exploit protein degradation pathways to complete their infection cycles. Zika virus (ZIKV) is an emerging orthoflavivirus causing serious neurologic disorders in neonates (congenital microcephaly) and adults (Guillain–Barré syndrome). Non-structural protein 5 (NS5), the largest and most conserved protein in the orthoflaviviruses, catalyses the synthesis and capping of new viral genomes. In addition to viral RNA replication in the cytoplasm, ZIKV NS5 is translocated into the nucleus to interfere with host antiviral responses. Here, we demonstrate that ZIKV NS5 co-immunoprecipitates with cellular UBA1. Immunofluorescence assays suggest that this interaction takes place primarily in the nucleus of an infected cell, although colocalization of both proteins is also detected in the cytosol. RNA interference-mediated depletion of UBA1 leads to reduced virus titres in the infected cells, while transient overexpression of UBA1 favours faster replication kinetics, with higher virus titres and protein levels detected. Moreover, UBA1-targeting drugs cause significant drops in virus infectivity. These results support a proviral role for UBA1 during ZIKV infection and encourage the potential use of inhibitors against this enzyme or its NS5-interacting epitopes as potential therapeutic targets.

## Introduction

Zika virus (ZIKV) is an emerging arbovirus, which has caused large outbreaks during the last decade. Estimates suggest that several million people were infected in the Americas during the 2015–2016 epidemic [1,2]. While most cases are asymptomatic, ZIKV infection during pregnancy can lead to severe congenital development defects, i.e. microcephaly, which cause profound cognitive and behavioural impairments in newborns, who consequently require long-term surveillance [3,4]. ZIKV infection has also been linked to serious neurological disorders in adults, with a significant number of people developing Guillain–Barré syndrome [5]. ZIKV belongs to the genus *Orthoflavivirus* within the *Flaviviridae* family [6]. While generally confined to tropical and subtropical regions, the global incidence and geographic expansion of ZIKV and other orthoflaviviruses causing severe human disease have been steadily growing in recent years. Among the most relevant orthoflaviviruses to public health are dengue (DENV), yellow fever and Japanese encephalitis viruses. All three combined are responsible for hundreds of thousands of cases of severe disease and tens of thousands of deaths annually [7,8]. In addition to human pathogens, other viruses having birds as their natural vertebrate hosts can also infect and lead to severe disease in humans. Neuroinvasive infection by West Nile virus (WNV) can cause a range of disorders of varying severity, such as acute flaccid paralysis, meningitis and encephalitis, with tens to hundreds of fatalities associated in the EU and the USA every year [8]. Usutu virus (USUV) is a new emerging orthoflavivirus, which has caused large epidemics in bird populations across Europe, with high mortality rates reported in some species [9,10]. While severe disease associated with USUV is rare in humans, a growing number of neurological disorders have been documented, suggesting that this pathogen may pose a new risk to public health [10,11].

The orthoflaviviruses are positive-stranded RNA viruses, containing a genome of ~11 kb in length. Once released in the cytoplasm, the viral genome is directly translated into a viral polyprotein by the ribosome [12]. Cellular and viral proteases cleave this precursor into three mature structural proteins (C, E and M) and seven non-structural proteins (NS1, NS2A, NS2B, NS3, NS4A, NS4B and NS5). NS5 is the largest and most conserved orthoflaviviral protein and contains two major catalytic activities: an RNA-dependent RNA polymerase (RdRp) domain in its C-terminus and a methyltransferase (MTase) in the N-terminus domain [13,14]. Viral genome replication takes place in invaginations of the endoplasmic reticulum (ER) where, in a coordinated manner with the remaining non-structural proteins, the RdRp domain synthesizes new viral genomes that are then capped by the MTase domain [12].

Viral replication and translation typically affect cellular protein homeostasis, and, as a consequence, diverse responses to stress are activated, including the unfolded protein response and protein degradation pathways [15,16]. Ubiquitin tagging is utilized in two major proteolytic pathways, the ubiquitin-proteasome system (UPS) and selective autophagy [17,18]. In the UPS, ubiquitinated proteins are degraded by the proteasome in a process assisted by the valosin-containing protein (VCP), also known as p97 [19]. In addition to protein degradation, an ample variety of cellular processes are regulated by ubiquitylation, such as controlling cell cycle progression, DNA damage repair, transcription, translation, vesicle transport and apoptosis [18]. Ubiquitylation is accomplished by a cascade of three enzymatic activities (E1, E2 and E3). While there are ~50 E2 and ~700 E3 different enzymes, the human genome only encodes 2 E1 proteins, stimulating the identification of E1-specific drugs to treat ubiquitylation-associated diseases [20]. Specifically, the first step during the ubiquitylation cascade has been postulated as an attractive target for the control of different types of cancer. Several drugs are currently being clinically trialled as potential chemotherapeutics [21,22].

Ubiquitin-like modifier-activating enzyme 1 (UBA1) is the major E1 enzyme in humans, playing a central role in protein homeostasis, while UBA6, the other human E1 enzyme, is restricted to a subset of cellular proteins and processes [21,22]. Two main isoforms of UBA1 are expressed as a result of alternate translational initiation sites (ATG), being UBA1a 40 aas larger than UBA1b. The additional N-terminal region in UBA1a contains a nuclear localization signal and four phosphorylation sites (absent in UBA1b), which modulate its biological functions in the nucleus [18]. During ubiquitylation, two ubiquitin molecules are loaded on UBA1. One is non-covalently bound to the active adenylation site (AAD), which is activated by adenylation. The other ubiquitin residue is covalently linked to the catalytic cysteine domain, from which is transferred to an E2 enzyme [21].

Here, we describe that human UBA1 is an interacting factor of ZIKV NS5 and plays a proviral role during virus replication. We found that both proteins pull down together in co-immunoprecipitation (co-IP) assays and colocalize in the nucleus and the cytoplasm of infected cells. Downregulation of UBA1 activity, either by small molecules targeting the enzyme or silencing its translation by RNA interference (RNAi) methods, led to decreases in the virus titres. Meanwhile, UBA1 overexpression led to higher viral loads.

## Methods

### Cells

African green monkey kidney epithelial (Vero) cells were grown in high-glucose Dulbecco’s modified Eagle’s medium (DMEM; Gibco) containing 5% (v/v) FBS (Sigma-Aldrich), 100 units ml^−1^ penicillin-streptomycin (PenStrep, Gibco) and 1 mM HEPES (Gibco). A549 cells, obtained from a human lung adenocarcinoma, and HEK 293 T cells, derived from human embryonic kidney cells, were maintained in high-glucose DMEM containing 10% (v/v) FBS, 100 units ml^−1^ penicillin-streptomycin and 2 mM l-glutamine (Gibco). All cell lines were maintained at 37 °C in the presence of 5% CO_2_ and high relative humidity.

### Virus strains and titration assays

The origin of ZIKV (PRVABC59, Puerto Rico, 2015) and USUV (Austria, 2001) has been described in previous manuscripts [23,25]. Virus titres in the supernatants collected from infected cells were determined by TCID_50_ assays, following the protocols previously described [25]. To this assay, 10^4^ Vero cells in 60 µl of media containing 5% FBS were added to a 96-well plate. The next day, 100 µl of tenfold serial dilutions of each virus in media containing 1% FBS was applied to wells, reaching a final volume of 160 µl. The dilution at which 50% of the cell cultures are infected, showing an apparent cytopathic effect under the microscope, is used to calculate the number of TCID_50_ per millilitre, using the protocols previously described [23,26].

### Virus inhibition assays by UBA1-targeting drugs

PYR-41, also known as 4[4-(5-nitro-furan-2-ylmethylene)-3,5-dioxo-pyrazolidin-1-yl]-benzoic acid ethyl ester, and TAK-243, also known as (1R,2R,3S,4R)-2,3-dihydroxy-4-[[2-[3-[(trifluoromethyl)thio]phenyl]pyrazolo[1,5-a]pyrimidin-7-yl]amino]cyclopentyl]sulfamic acid methyl ester, were purchased from Selleckchem. To determine the 50% inhibitory concentration (IC_50_) for each drug, we seeded 2×10^4^ Vero cells in each well of a 96-well plate. The next day, the supernatant was removed, and the cells were washed in fresh media containing 1% FBS. The virus was then applied at the m.o.i. indicated, and adsorption was allowed for 1 h at 37 °C with 5% CO_2_. The inoculum was then removed, and the cells were washed with media to eliminate unattached virus. Finally, the cells were covered in media containing 1% FBS and in the presence or absence of UBA1 drugs. Supernatants collected at 24 and 48 h post-infection were titrated as explained above.

### UBA1 protein knockdown by RNAi

UBA1 protein expression was knocked down with a specific Dicer-substrate small interfering RNA (DsiRNA) duplex from Integrated DNA Technologies (IDT), targeting the region corresponding to exon 14 in the mRNA (IDT reference hs.Ri.UBA1.13.1, target sequence 5′-AGAAGUCAAAUCUGAAUCGACAGUUUC-3′). As a negative control, an unspecific DsiRNA (predicted not to recognize any target sequence in the human genome) was used (IDT reference 51-01-14-03, antisense sequence 5′-AUACGCGUAUUAUACGCGAUUAACGAC-3′). To silence UBA1 expression, a total of either 3 or 10 pmol of each DsiRNA were added into each well of a 96-well plate, in two consecutive transfection rounds (either 1.5 or 5 pmol in each), in the presence of Lipofectamine RNAiMAX (Invitrogen) and Opti-MEM (Gibco), as suggested by the manufacturers. In the first transfection step, the DsiRNA-Lipofectamine complexes were prepared in 50 µl of Opti-MEM and applied to an empty 96-well plate. After 20 min, 1.5×10^4^ A549 cells were seeded on top. Twenty-four hours later, the cells were subject to a second DsiRNA transfection. To this, cellular supernatants were removed, 100 µl of fresh media was added and the DsiRNA-Lipofectamine complexes in 50 µl of Opti-MEM were applied to the monolayers.

### Virus infections in UBA1-depleted cells by DsiRNAs

Twenty-four hours after the second DsiRNA transfection, the cellular supernatants were removed, and the virus was inoculated at an m.o.i. of 0.1 TCID_50_/cell. Following virus adsorption during 1 h at 37 °C, the inoculum was removed and replaced with fresh media containing 1% FBS. Aliquots from the infected cultures were collected at different time points, and virus titres were determined by TCID_50_ assays as detailed above.

### Cell viability assays

To quantitate the amount of metabolically live cells after exposure to either UBA1 drugs (PYR-41 and TAK-243) or DsiRNA molecules (either targeting UBA1 mRNA transcripts or the unspecific control), we used the CellTiter-Blue Cell Viability Assay (Promega), following the protocols previously described in our laboratory [25,27]. The viability values in each assay are calculated as a percentage of the fluorescence emitted by mock-treated cultures carried out in parallel.

### Reverse transcription-quantitative PCR (RT-qPCR) assays for the detection of UBA1 mRNA transcripts

To confirm UBA1 expression knockdown after DsiRNA transfection, A549 cells were collected at 48 h after the second transfection cycle in the lysis buffer provided in the NZY Total RNA Isolation kit (NZYTech). RNA was then extracted following the instructions provided by the manufacturer. RNA levels of target host factors were analysed by using the One-step NZYSpeedy RT-qPCR Green kit with ROX (NZYTech) in an Applied Biosystems 7500 Real-Time PCR thermocycler using specific primer pairs for UBA1 or a housekeeping gene (GAPDH), both provided by IDT (reference numbers: 239200034 and 238397099, respectively). Expression levels were then compared and relativized to untransfected cells. The efficiency of UBA1 transcript downregulation was calculated following the 2^-ΔΔCT^ method [28].

### Vectors for protein expression and protocols for transfection

Protocols for the expression of HA-tagged ZIKV, USUV and WNV NS5 proteins in mammalian cells and His-tagged ZIKV NS5 in *Escherichia coli* have been previously described [27]. To prepare constructs for UBA1 expression in mammalian cells, we first extracted whole-cell RNA from human A549 cultures, using the GeneJet RNA Purification Kit (Thermo Fisher Scientific), as suggested by the manufacturer. The cellular RNA was then reverse transcribed into cDNA, using random hexamers and SuperScript III (Invitrogen), following the protocols specified by the provider. The UBA1 transcript was then amplified using the Q5 High-Fidelity DNA Polymerase (New England Biolabs) and specific primers. For the untagged UBA1 expression vector (pUBA1), a forward primer with sequence 5′-GCATTGAAGCTTGCCACCATGTCCAGCTCGCCGCTGTCCAAG-3′ (containing a HindIII restriction site and a Kozak sequence preceding 24 nts spanning the 5′-end of UBA1a ORF) and a reverse primer with sequence 5′-CTAGAGGAGGATATCCCATCAGCGGATGGTGTATCGGACATAG-3′ (containing an EcoRV cloning site and a stop codon, followed by a 22-nt sequence complementary to the 3′-end of UBA1 coding region) were used. For the preparation of a construct expressing a FLAG-tagged version of UBA1 (pFLAG-UBA1), the same forward primer as above but containing a FLAG-coding sequence between the ATG and the UBA1 5′-end (5′-GCATTGAAGCTTGCCACCATGGACTACAAAGACGATGACGACAAGTCCAGCTCGCCGCTGTCCAAG-3′) was used. The same reverse primer was used for the generation of pFLAG-UBA1 than for pUBA1. The amplification product and the pcDNA3.1 vector (Invitrogen) were both digested with HindIII and EcoRV and, after gel band purification, ligated and transformed into *E. coli* competent cells. Sanger sequencing was used to assess whether the constructs contained the expected sequences.

To the expression of recombinant NS5s and UBA1 proteins in mammalian cells, 5×10^6^ 293 T cells were seeded into 10 cm diameter plates, and the corresponding plasmid was transfected the same day, using Lipofectamine 3000 as recommended by the provider (Invitrogen). Briefly, 20 µg of each plasmid was diluted in 250 µl of Opti-MEM (Gibco), and then, 20 µl of P3000 reagent was added to the mix. In a different tube, 20 µl of Lipofectamine 3000 was diluted in 250 µl of Opti-MEM. Both mixes were combined and incubated at room temperature for 15–20 min, and the complexes were applied dropwise to each dish. At 42 h post-transfection, the cells were washed twice in 1× Dulbecco’s PBS (1× DPBS, Sigma-Aldrich) and collected for subsequent analysis. Plasmid pEYFP-N1 (Clontech) encoding the enhanced yellow fluorescent protein (EYFP) was used as a transfection control in different assays indicated across the text.

### Virus infections in cells overexpressing UBA1

To examine the impact of UBA1 overexpression on ZIKV replication, 1×10^5^ 293 T cells were seeded in each well of a 24-well plate. The next day, 1 µg of pUBA1, pFLAG-UBA1 or empty vector (pcDNA3.1) was transfected into each well, using Lipofectamine 3000 and protocols suggested by the manufacturer. At 24 h post-transfection, cell supernatants were removed and cells were infected with ZIKV at an m.o.i. of 3 TCID_50_/cell. After 1 h to allow adsorption, the inoculum was removed, the cells were washed and 1 ml of fresh media containing 1% FBS was added. Cellular supernatants were collected at different time points to analyse virus titres by TCID_50_ assays (above). Cell monolayers were collected in lysis buffer (10 mM Tris HCl pH 7.5, 150 mM NaCl, 0.5 mM EDTA, 0.1% SDS, 1% Triton™ X-100 and 1% deoxycholate) in the presence of 1× Halt protease inhibitor cocktail (Invitrogen) and were processed for subsequent Western blot (WB) analysis.

### Co-IP assays

Immunoprecipitations of proteins containing an HA-epitope tag were performed using anti-HA agarose beads as suggested by the manufacturer (Pierce). Briefly, cells transfected with constructs expressing HA-tagged NS5s were washed twice in 1× DPBS and then collected in lysis buffer in the presence of 1× protease inhibitor cocktail (as mentioned above in ‘Virus infections in cells overexpressing UBA1’) and 250 units ml^−1^ of benzonase nuclease (Sigma-Aldrich). Next, protein concentration was normalized in each lysate after quantification with the BCA Protein Assay Kit (Pierce) and then diluted in 1.5 vols of dilution buffer (10 mM Tris-Cl pH 7.5, 150 mM NaCl and 0.5 mM EDTA) to a final volume of 1 ml. Lysates were then incubated overnight in the presence of 40 µl of anti-HA bead slurry, previously equilibrated in dilution buffer, on a rotary shaker at 4 °C. Cell supernatants were then removed, and the beads were washed four times in wash buffer (dilution buffer containing 0.05% Nonidet P40 substitute). The beads were then resuspended in 60 µl of 1× NuPAGE LDS buffer (Invitrogen) and heated at 90 °C for 10 min before loading onto an SDS-PAGE gel.

### WB assays

To study the host and virus protein level kinetics during infection, we seeded 2×10^6^ A549 cells on 10 cm diameter plates. The next day, cells were infected with ZIKV or USUV at an m.o.i. of 3 TCID_50_/cell, and the cells were collected at different time points. To detect the transiently expressing recombinant proteins (HA-NS5, UBA1 or FLAG-UBA1), the cells were transfected and later processed as suggested above (section entitled ‘Vectors for protein expression and protocols for transfection’). To assess protein knockdown efficacy in cells transfected with DsiRNAs, we followed the protocols described above.

The cells were washed twice in 1× DPBS and then collected in lysis buffer as mentioned above. Cell lysates were then spun at 14,000 r.p.m. for 10 min at 4 °C, and the supernatant was transferred to a new tube. Protein concentration in lysates was normalized after quantification, using the BCA Protein Assay Kit (Pierce), as explained above. Then, 50 µg of each sample was resolved in SDS-PAGE electrophoresis and transferred to an Immobilon-P PVDF membrane (Millipore), using the Power Blotter Cassette (Invitrogen). The membranes were blocked in 10% non-fat dry milk in 1× PBS containing 0.05% Tween-20 (PBS-T) during 1 h at room temperature. Then, the membranes were incubated in the presence of the corresponding primary antibody (1:500 dilution in 4% non-fat dry milk), washed thrice in PBS-T and then incubated in the presence of a 1:4000 dilution of either HRP-conjugated anti-rabbit (Invitrogen, reference 31466) or anti-mouse antibodies (Cell Signaling, reference 7076), following the protocols previously described [27]. The blot was developed using the SuperSignal West Pico Plus Chemiluminescent Substrate Kit (Thermo Fisher Scientific). Positive bands were detected in an ImageQuant LAS 4000 device (GE Healthcare Bio-Sciences, Uppsala, Sweden). Primary antibodies used for WB are rabbit anti-UBA1 (Invitrogen, MA5-35786), mouse anti-HA epitope (BioLegend, reference 901501), rabbit anti-FLAG epitope (Proteintech, reference 20543–1-AP) and rabbit anti-ZIKV NS5 (GeneTex, GTX133328). As loading controls, HRP-conjugated antibodies against GAPDH (GeneTex, GTX627408-01) and tubulin (Cell Signaling, 12351S), not requiring secondary antibody to detection, were also used.

### Immunofluorescence assay and confocal microscopy

To determine the subcellular distribution of ZIKV NS5 and UBA1 in the infected cell, 1×10^5^ A549 cells were seeded on a 12 mm diameter coverslip placed into a 24-well plate. The cells were then infected at an m.o.i. of 3 TCID_50_/cell, and at different time points, the cultures were washed in 1× PBS and fixed in 4% paraformaldehyde. The plates were then processed following the immunocytochemistry protocols previously described in our laboratory [29], using mouse anti-UBA1 (Santa Cruz, sc-53555), rabbit anti-UBA1 (Invitrogen, MA5-35786), rabbit anti-ZIKV NS5 (GeneTex, GTX133328) and mouse anti-dsRNA (MUbio, reference 10010200) at a 1:500 dilution. As secondary antibodies, we used Alexa Fluor 546 donkey anti-mouse IgG (A10036, Invitrogen) and Alexa Fluor 488 chicken anti-rabbit IgG (A21441, Invitrogen). Nuclei were then stained with DAPI, and after washing in 1× PBS, the cells were mounted using Fluorescent Mounting Medium (Dako). Images were then analysed in a ZEISS LSM 710 confocal microscope and acquired with a format of 8-bit depth 1024×1024 using an oil immersion Plan-Apochromat 63×/1.40 Oil DIC M27 objective. Three channels were sequentially registered to capture Alexa Fluor 546 (channel 1:555 nm laser source and collecting 550–720 nm wavelength light), EGFP-T2 (channel 2:488 nm laser source and collecting 495–550 nm wavelength light) and DAPI (channel 3:405 nm laser source and collecting 427–494 nm wavelength light).

### Statistical analysis

Statistical significance was determined using GraphPad Prism 8 and different statistical tests as specified in each corresponding figure legend.

## Results

### UBA1 interacts with different orthoflaviviral NS5 proteins in co-IP assays

**Fig. 1.**
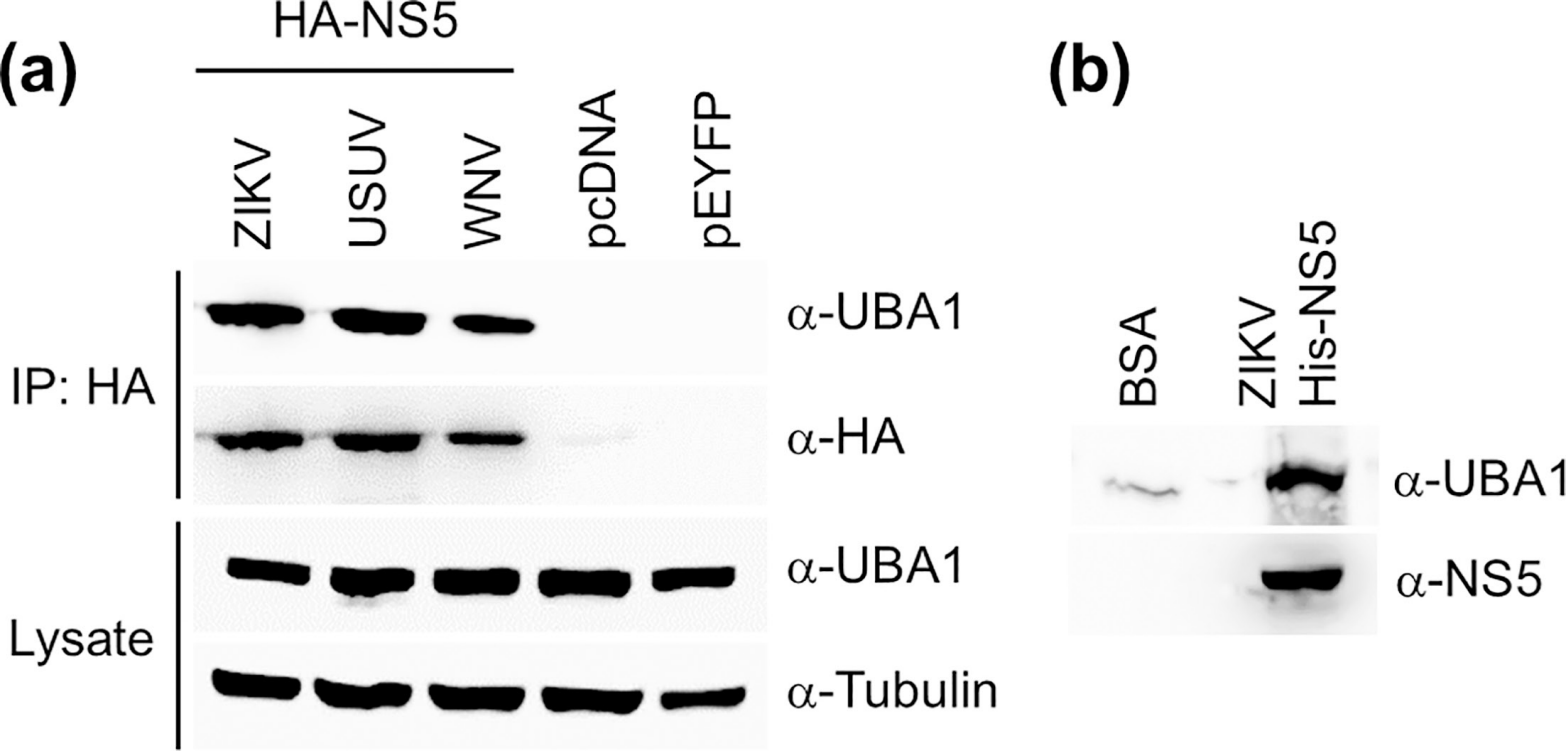
UBA1 interacts with orthoflaviviral NS5 proteins. (**a**) UBA1 is co-immunoprecipitated with ZIKV, USUV and WNV NS5 proteins. 293 T cells were transfected with constructs expressing HA-tagged versions of each NS5 (ZIKV, USUV and WNV), EYFP (pEYFP-N1, shown as pEYFP) or an empty vector (pcDNA3.1, shown as pcDNA). NS5 proteins were immunoprecipitated with anti-HA agarose beads. (**b**) UBA1 coprecipitated with recombinant His-tagged ZIKV NS5 in assays using Ni-NTA agarose resin (Invitrogen). For the assay, 3 mg of whole-cell protein, extracted from 293 T cells, was incubated in the presence of either 90 µg of recombinant BSA or His-tagged ZIKV NS5.

Preliminary data in our group suggested that UBA1 could be an interacting host factor of the RdRp domain (residues 276 to 903) in ZIKV NS5 (Fig. S1, available in the online version of this article). In agreement with this observation, UBA1 was identified as an interacting factor of ZIKV NS5 in previous studies, although no further analysis regarding this interaction followed [30]. To further confirm these previous observations, we examined whether the two proteins interacted by co-IP assays, using as a bait a construct encoding the full-length ZIKV NS5 fused to an HA-tag in its N-terminus (Fig. 1a). Endogenous UBA1 was positively enriched in pulldowns of ZIKV HA-NS5 recombinantly expressed in 293 T cells (>100-fold) when compared to controls transfected with either an empty vector (pcDNA3.1) or a plasmid expressing EYFP (pEYFP). To further investigate whether UBA1 also interacted with other orthoflavivirus NS5s, we used constructs expressing the corresponding proteins encoded by WNV and USUV (Fig. 1a), which are readily available in our group [27]. We confirmed that UBA1 positively co-immunoprecipitated with all three different NS5s (UBA1 >100-fold higher in NS5-containing samples than in controls), suggesting that this interaction may be relevant to the orthoflavivirus life cycle. As expected, UBA1 was not found in a control co-IP, using HA-tagged LacZ (Fig. S2a). We further demonstrated that NS5-UBA1 is a specific interaction, and not a consequence of unspecific binding to nuclear proteins, by examining the presence of TBP, an unrelated nuclear protein (Fig. S2a).

To obtain additional confirmation for this interaction, we used recombinant His-tagged ZIKV NS5 and Ni-NTA beads in coprecipitation assays, using whole-cell lysates from 293 T cells as a source for host cell proteins. We found >4 times larger amounts of UBA1 in samples incubated in the presence of His-NS5 than in controls (Fig. 1b). UBA1 was not detected when an unrelated His-tagged protein was used as a bait (Fig. S2b). These results support that ZIKV and other orthoflaviviral NS5s interact with host cellular UBA1 in cell culture.

### UBA1 expression is stimulated in ZIKV-infected cells

**Fig. 2.**
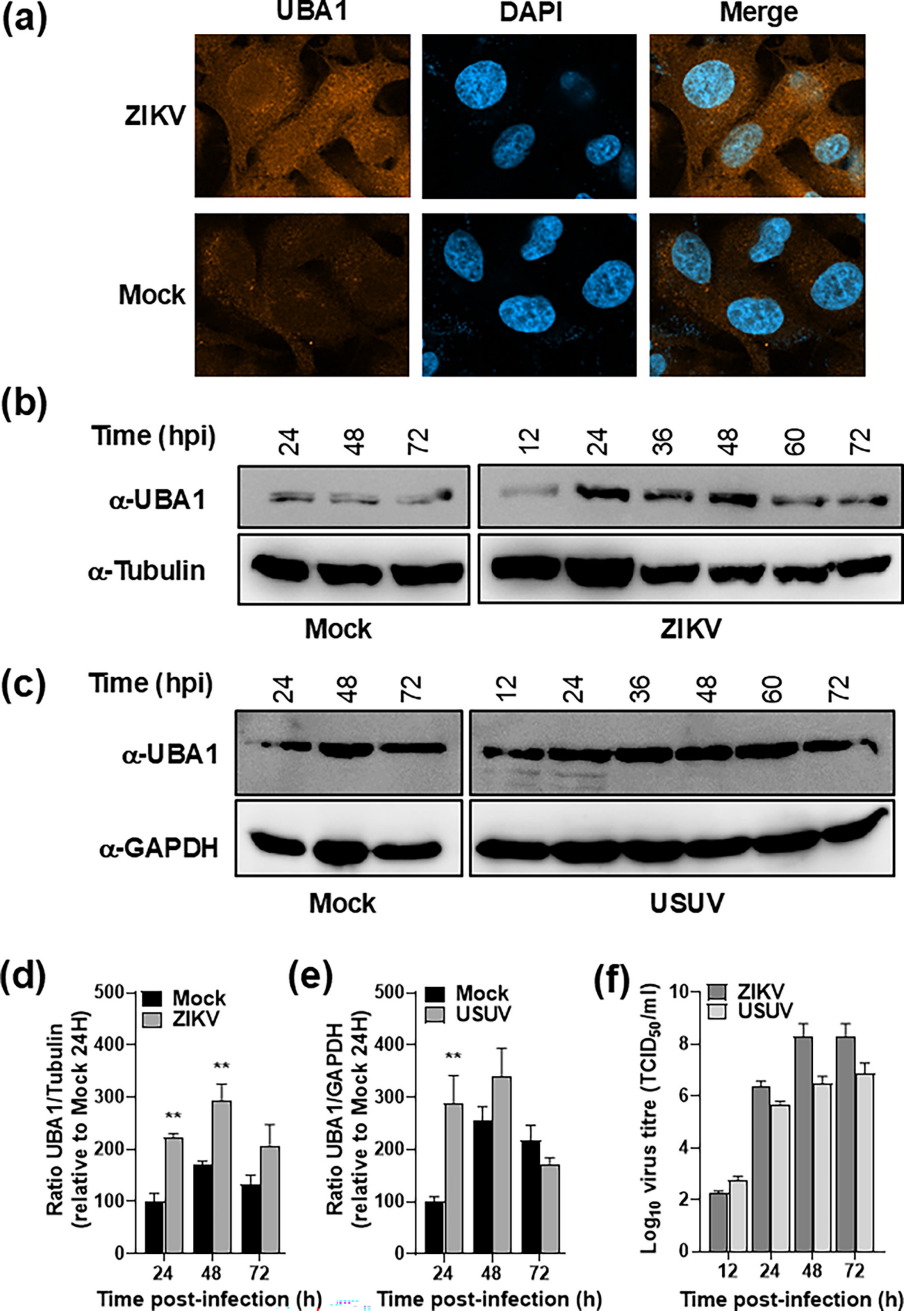
Increased UBA1 protein levels in ZIKV- and USUV-infected cells. (**a**) Immunofluorescence assay (IFA) to the detection of UBA1 in mock or ZIKV-infected A549 cells. (b–e) UBA1 protein levels detected in A549 cell lysates collected at different time points in mock and infected cultures. (**b, c**) Representative blot of UBA1 kinetics in cells infected with either ZIKV (b) or USUV (c). (**d, e**) UBA1 protein level kinetics in cells infected with either ZIKV (**d**) or USUV (**e**). Each value in the graph is the average of three independent biological replicas (*n*=3). Bars represent the sem for each data point. Significant differences between mock and infected samples are indicated (two-way ANOVA, Sidak’s multiple comparison test; *P<0.05; **P<0.01). (**f**) Virus titre kinetics in the supernatant of A549 cells infected with either ZIKV (dark grey) or USUV (light grey).

Initial confocal analyses suggested that UBA1 expression was significantly increased in ZIKV-infected cells compared to mock-infected cells (Fig. 2). To assess this possibility, we quantified UBA1 protein levels in ZIKV- and USUV-infected cells by WB at different times post-infection (Fig. 2b, c). We confirmed that UBA1 protein expression levels were significantly larger at 24 h post-infection (>2-fold), both in cells infected with ZIKV or USUV, and at 48 h post-infection in ZIKV-infected cells (Fig. 2d, e). Larger amounts of UBA1 levels detected at 24 and 48 h post-infection correlate with elevated virus titres in the cellular supernatants (Fig. 2f). These results suggest that the expression of host genes implicated in protein ubiquitylation pathways may be positively affected by orthoflavivirus replication.

### ZIKV NS5 colocalizes with UBA1 in an infected cell

**Fig. 3.**
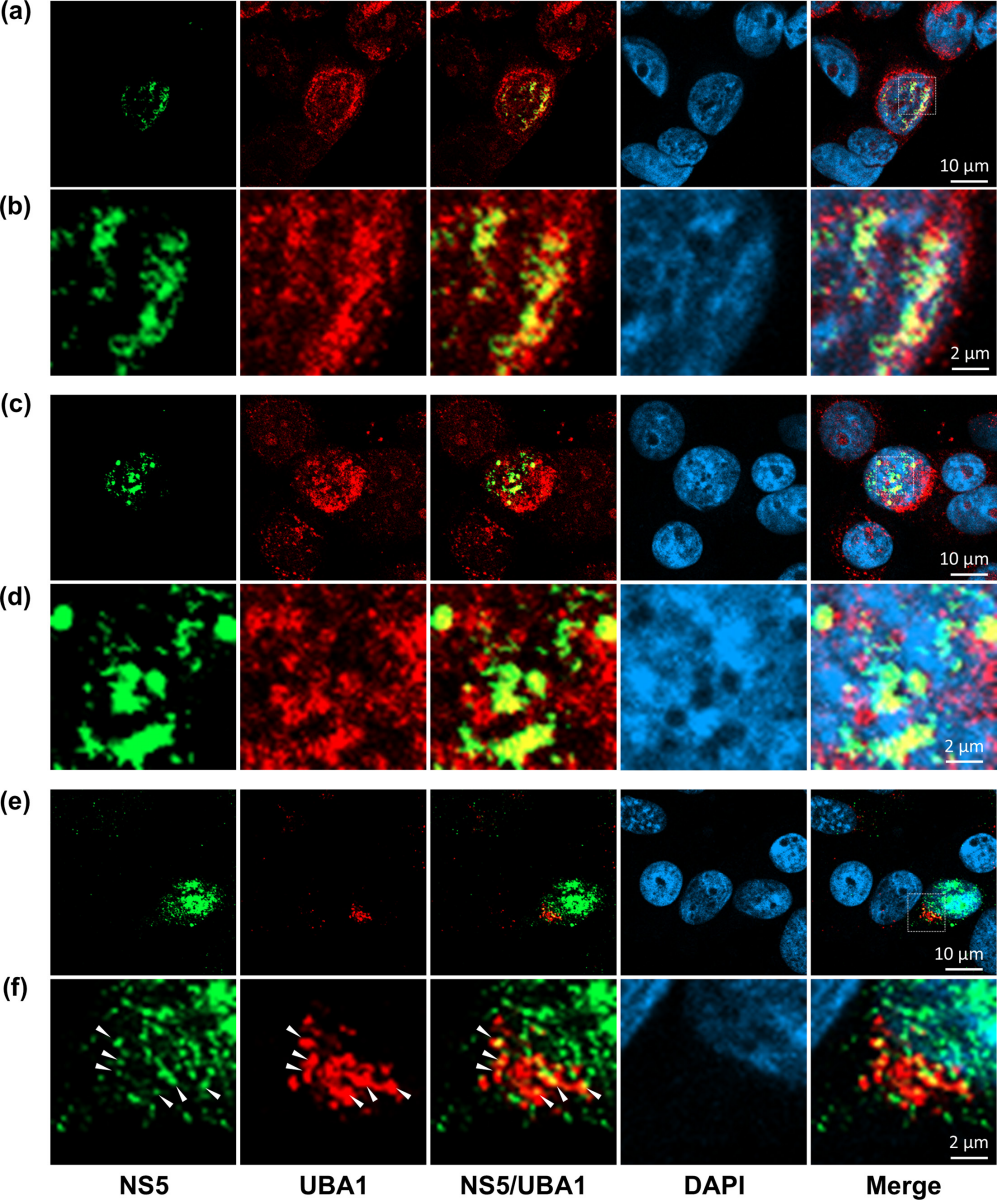
ZIKV NS5 colocalizes with UBA1 in the infected cell. Fluorescence signals emitted by UBA1 and NS5 overlap in nuclear and cytoplasmic regions of the infected cells at 36 h post-infection (merge). To detect NS5 (green) and UBA1 (red), specific rabbit anti-NS5 and mouse anti-UBA1 antibodies were used. Nuclei were stained with DAPI (blue). All signals combined are represented (merge). (**a, c**) UBA1 and NS5 are detected in the nuclei of ZIKV-infected cells. (**b, d**) Close-up view of the areas where colocalization between UBA1 and NS5 was observed. An amplification of the image selected in the dotted square area in (**a**) and (c) is provided in (b) and (**d**), respectively. (**e**) Colocalization of NS5 and UBA1 in the cytoplasmic region of A549 cells. (**f**) An amplification of the image selected in the dotted square area in (**e**) is provided. Arrows indicate those regions where colocalization is detected.

**Fig. 4.**
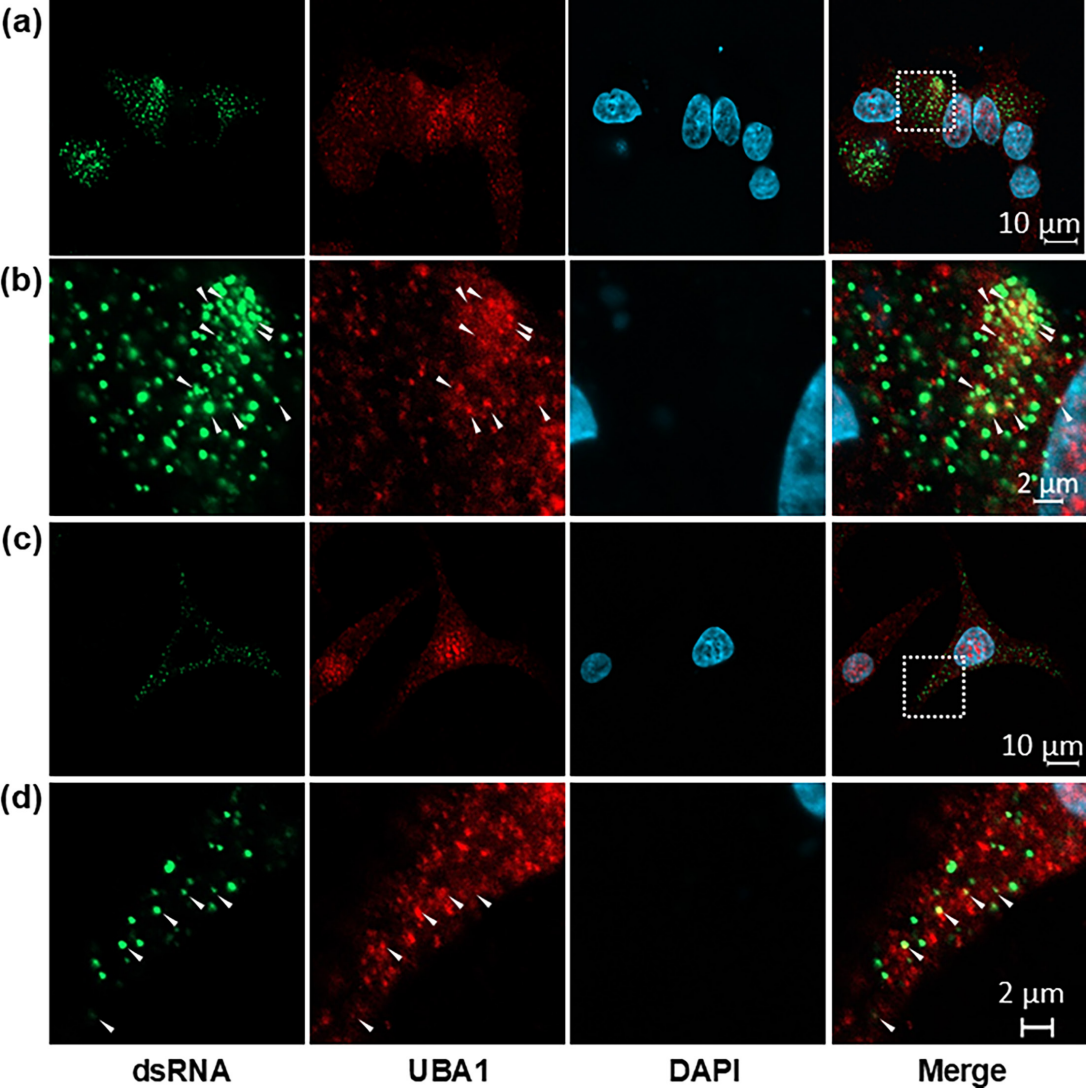
UBA1 colocalizes with dsRNA in ZIKV-infected cells. Fluorescence emitted by UBA1 and dsRNA overlap in the cytoplasm of A549 cells at 48 h post-infection. To detect dsRNA (green) and UBA1 (red), specific mouse anti-dsRNA and rabbit anti-UBA1 antibodies were used. Nuclei are stained with DAPI (blue). All signals combined are represented (merge). (**a, c**) UBA1 and dsRNA are detected in the cytoplasm of ZIKV-infected cells. (**b, d**) Close-up view of the areas where colocalization between UBA1 and dsRNA was observed. An amplification of the image selected in the dotted square area in (**a**) and (**b**) is provided in (**c**) and (**d**), respectively. Arrows indicate those regions where colocalization is detected.

To visualize the interaction between ZIKV NS5 and UBA1 at the cellular level, we analysed their expression and localization in infected cells by confocal microscopy. Although viral replication occurs in the cytoplasm, in most cells, the vast majority of ZIKV NS5 concentrates in the nucleus, in agreement with previous observations [31,32]. Meanwhile, we found that UBA1 was evenly distributed across the cytoplasm and the nucleus of both ZIKV- and mock-infected cells (Fig. 3). At 36 h post-infection, UBA1 and NS5 colocalization could be detected in the nuclei of infected cells (Fig. 3a–d). ZIKV NS5 and UBA1 also colocalized in the nuclei of cells transfected with constructs expressing HA-tagged ZIKV NS5, further confirming the interaction between both proteins (Fig. S3). In a minority subset of ZIKV-infected cells where NS5 was primarily located in the cytosol, we also observed that both protein signals overlapped (Fig. 3e, f). Consequently, since ZIKV NS5 is predominantly nuclear, the colocalization of UBA1 with NS5 in cytoplasmic replication factories could have been overlooked in most cells. To further explore whether UBA1 could be associated with viral genome synthesis, we investigated the intracellular localization of viral RNA intermediate replication complexes using a specific antibody against dsRNA, as specified in Methods. We found that dsRNA followed a dotted pattern across the cytoplasm with some of these dots colocalizing or in close proximity to UBA1-positive regions (Fig. 4). All these data support that UBA1 and NS5 interact in a ZIKV-infected cell, suggesting a biological role for UBA1 during the virus life cycle.

### ZIKV and USUV replication is abrogated in UBA1-depleted cells

**Fig. 5.**
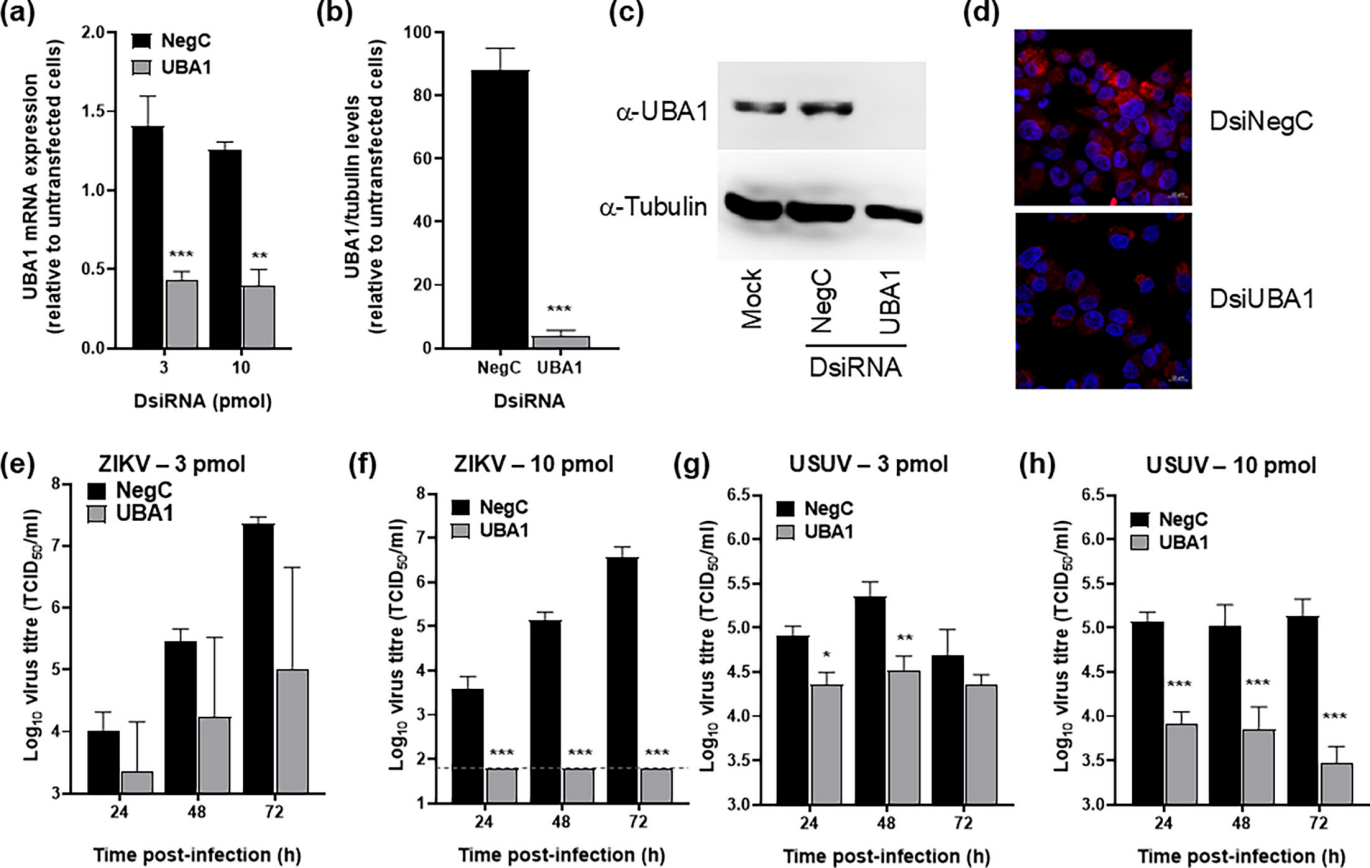
ZIKV and USUV replication is restricted in UBA1-depleted cells. (**a**) RT-qPCR analysis of UBA1 expression in A549 cells transfected with either a UBA1-targeting or a non-human target DsiRNA (NegC). UBA1 RNA expression was normalized to GAPDH mRNA levels. Values obtained are the average of four independent biological replicas (*n*=4), and relative to untransfected cells carried out in parallel. Significant differences observed between samples are indicated (two-way ANOVA, Sidak’s multiple comparisons test; **P<0.01; ***P<0.001). (**b, c**) UBA1 protein levels detected by WB in A549 cells previously transfected with either a specific (UBA1) or a non-human target (NegC) DsiRNA. Protein levels were normalized to the detection of tubulin. (**b**) Values are the average of three independent experiments (*n*=3) and represented as a percentage of those observed in untransfected cells. A significant difference between samples was observed (*t*-test; ***P<0.001). (**c**) Representative WB of UBA1 protein levels in cells transfected with DsiRNAs. (**d**) UBA1 detection by IFA in cells previously transfected with either a specific (DsiUBA1) or a non-human target (DsiNegC) DsiRNA. To detect UBA1 (red), a specific mouse anti-UBA1 antibody was used. Nuclei were stained with DAPI (blue). (e–h) Virus infectivity was examined by TCID_50_ assays of samples collected at different time points from cells previously transfected with 3 (**e, g**) or 10 pmol (**f, h**) of each DsiRNA, as indicated in (**a**). Twenty-four hours after the second transfection with DsiRNAs, cells were infected with ZIKV (**e, f**) or USUV (**g, h**) at an m.o.i. of 0.1 TCID_50_/cell. Each value in the graph is the average of at least three independent biological replicas (*n*≥3). Significant differences observed between unspecific and UBA1-targeting DsiRNA-transfected cells are indicated (two-way ANOVA, Sidak’s multiple comparisons test; *P<0.05; **P<0.01; ***P<0.001). Bars represent the sem for each data point.

To elucidate the possible contribution of UBA1 to the flavivirus life cycle, we investigated how its knockdown affected virus replication. To this aim, we used a specific DsiRNA, which recognizes both UBA1 transcript variants. We first confirmed that the transfection of DsiRNA targeting UBA1 resulted in decreased mRNA and protein levels by RT-qPCR, WB and immunofluorescence assay (IFAs) (Fig. 5). Transfection with an unspecific DsiRNA (NegC), which does not recognize any human sequence, did not affect UBA1 mRNA or protein levels by RT-qPCR or WB (Fig. 5a–c). Likewise, UBA1 protein levels were not affected by NegC (Figs 5d and S4). Cell viability was only modestly affected by UBA1 knockdown, with >80% of metabolically active cells in UBA1-downregulated cultures when compared to controls transfected with NegC (Fig. S4).

Depletion of UBA1 during infection caused significant decreases in ZIKV and USUV titres with respect to controls. We found >10- and 100-fold decreases in ZIKV titres at 48 and 72 h post-infection, respectively, in cells transfected with 3 pmol of UBA1-specific DsiRNA (Fig. 5e). No ZIKV infectivity could be rescued at any time point in cells transfected with 10 pmol (Fig. 5f). For USUV, we also observed significant decreases in the virus titres of infected cells previously transfected with either 3 or 10 pmol of UBA1-targeting DsiRNA (Fig. 5g, h). These data support that UBA1 is a proviral factor and its absence dramatically affects viral replication.

### UBA1 overexpression results in increased viral titres and protein levels

**Fig. 6.**
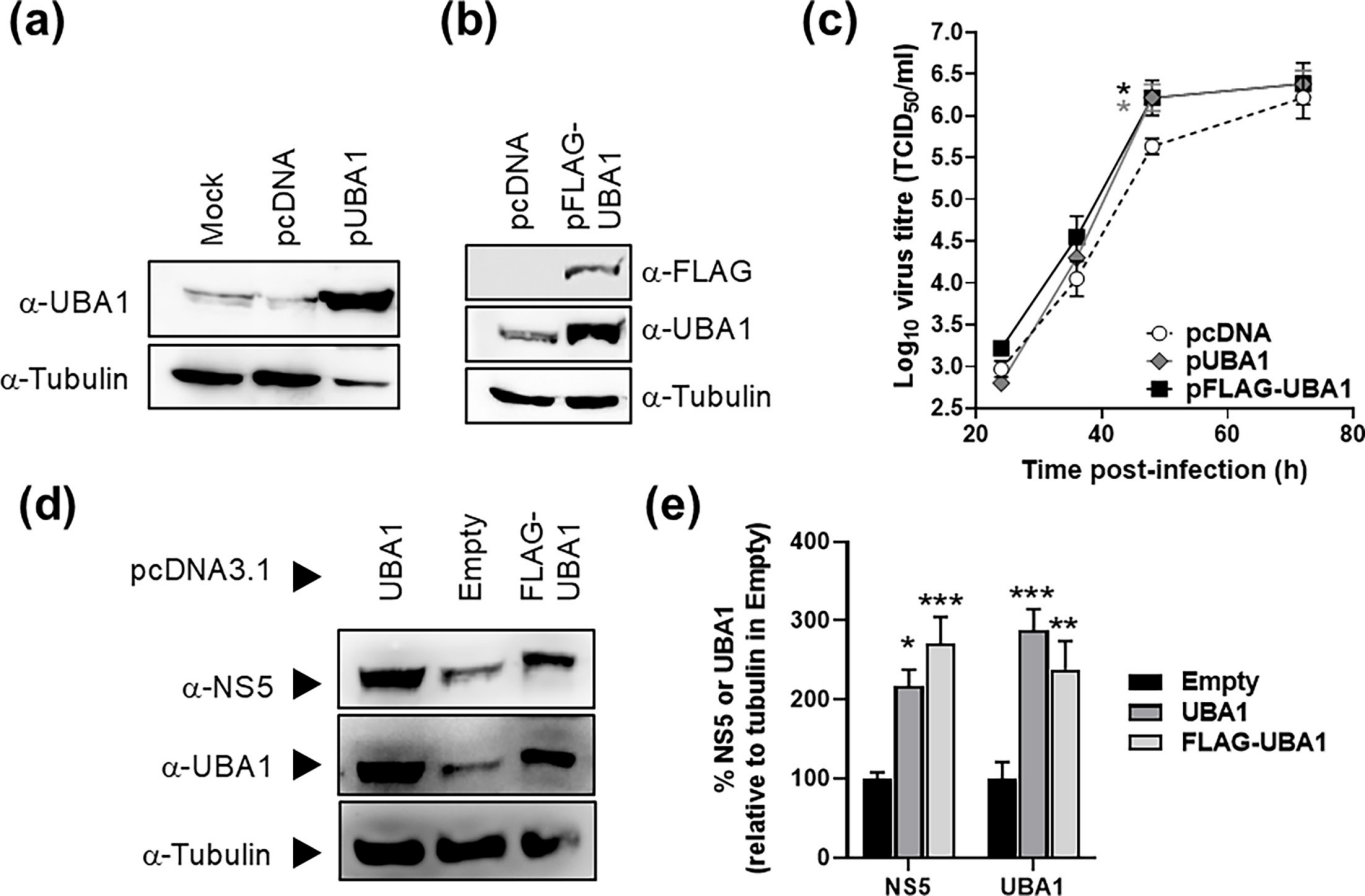
Enhanced ZIKV replication in cells overexpressing UBA1. (**a, b**) UBA1 is overexpressed in 293 T cells transfected with either pUBA1 (**a**) or pFLAG-UBA1 (**b**) when compared to untransfected cultures (mock) or cells transfected with an empty construct (pcDNA3.1, namely, pcDNA). (**a**) Larger levels of UBA1 detected in cells transfected with pUBA1; tubulin was used as a reference loading control. (**b**) Larger amounts of UBA1 detected in cells transfected with pFLAG-UBA1 when using a specific anti-UBA1 antibody. A positive band of the expected size for UBA1 was detected with an anti-FLAG antibody. Tubulin was used as a reference loading control. Representative images were included, which were obtained from two independent biological samples (two different WBs for each sample). (**c**) Virus titres obtained in cells previously transfected with pUBA1, pFLAG-UBA1 or an empty vector (pcDNA). Cells were infected with ZIKV at an m.o.i. of 1 TCID_50_/cell, and samples collected at different time points were titrated (four independent biological replicas). Significant differences with respect to mock-transfected cells (pcDNA) are indicated (two-way ANOVA, Dunnett’s multiple comparisons test; *P<0.05). (**d**) Representative WB of ZIKV NS5 and UBA1 protein levels in cells previously transfected (36 h earlier) with pUBA1, pFLAG-UBA1 or an empty vector (pcDNA); as a loading reference control, an anti-tubulin antibody was used (four independent biological replicas). (**e**) Ratio of NS5 and UBA1 protein levels relative to mock-transfected cells (empty vector) at 36 h post-infection. Tubulin was used as a reference control (four biological replicas).

To further examine whether UBA1 could be a positive factor intervening during orthoflaviviral replication, we examined the effect of its overexpression on ZIKV. To this aim, we prepared constructs expressing both FLAG-tagged and untagged versions of UBA1 as described in Methods (pFLAG-UBA1 and pUBA1, respectively). We first confirmed by WB that cells transfected with each of these constructs expressed higher UBA1 levels than untransfected cells (mock) or cells transfected with an empty vector (Fig. 6a, b). We found that both transfections of pUBA1 and pFLAG-UBA1 vectors resulted in a >3-fold increase in UBA1 levels when compared to controls. We confirmed with an anti-FLAG antibody that UBA1 overexpression in pFLAG-UBA1 samples correlated with the detection of a band of the expected size (Fig. 6b). To investigate the effect of UBA1 overexpression on ZIKV replication, we analysed the virus titres in cells previously transfected with either pUBA1 or pFLAG-UBA1. We observed a significant increase in ZIKV yields at 48 h post-infection in both cells overexpressing UBA1 (either native or FLAG-tagged), with respect to mock-transfected cells (Fig. 6c). We also found increased viral protein levels (NS5) in cells overexpressing UBA1 by WB at 36 h post-infection (Figs 6 and S5). The amount of ZIKV NS5 detected in cells transfected with pUBA1 and pFLAG-UBA1 was significantly higher (Fig. 6e), than in controls (empty vector). Altogether, these data support that UBA1 positively contributes to the ZIKV life cycle.

### Small molecules targeting UBA1 inhibit ZIKV and USUV infectivity

**Fig. 7.**
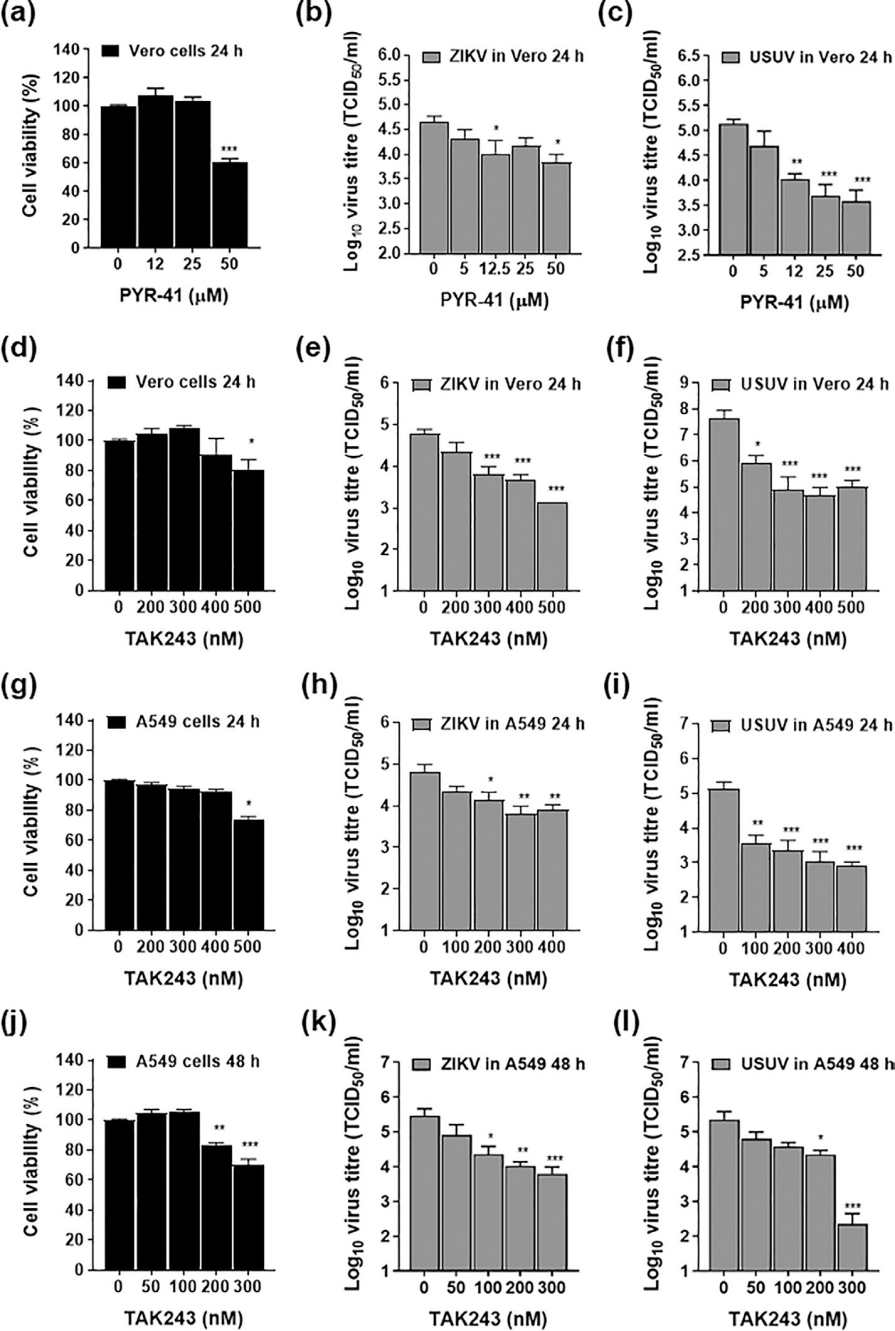
UBA1 drugs PYR-41 and TAK-243 inhibit orthoflaviviral replication in cell culture. (a–f) UBA1 drugs PYR-41 and TAK-243 inhibit ZIKV and USUV replication in Vero cells. (**a, d**) Relative number of metabolically live Vero cells after treatment with increasing concentrations of PYR-41 (**a**) and TAK-243 (**d**) during 24 h. The relative amount of metabolically active cells in treated samples is calculated by using the CellTiter-Blue Cell Viability Assay kit as suggested by the manufacturer (Promega). Values are represented as a percentage relative to mock-treated cells. (**b, c, e, f**) The antiviral activity was examined by TCID_50_ assays of samples collected from infected cells treated with increasing concentrations of drugs. To this, Vero cells were inoculated with either ZIKV at an m.o.i. of 0.2 TCID_50_/cell or USUV at 0.02 TCID_50_/cell. At 24 h post-infection, the cellular supernatants were collected and the virus titres were quantified. (**b, c**) Virus titres at 24 h post-infection in PYR-41-treated cells, previously infected with ZIKV (**b**) or USUV (**c**). (**e, f**) Virus titres at 24 h post-infection in TAK-243-treated cells, previously infected with ZIKV (**e**) or USUV (**f**). (g–l) UBA1 drug TAK-243 inhibits ZIKV and USUV replication in human A549 cells. (**g, j**) Relative number of metabolically active A549 cells after treatment with increasing concentrations of TAK-243 at 24 (**a**) and 48 (**d**) h. The relative number of viable cells is calculated as mentioned above. (**h, i, k, l**) The antiviral activity was examined by TCID_50_ assays of samples collected at 24 or 48 h post-infection from cells treated with increasing concentrations of TAK-243. The treated cultures were previously inoculated with either ZIKV or USUV at an m.o.i. of 0.1 TCID_50_/cell. (**b, e**) ZIKV titres at 24 (**h**) and 48 (**k**) h post-infection. (**i, l**) USUV titres at 24 (**i**) and 48 (**l**) h post-infection. Each value in the graph is the average of at least three independent biological replicas (*n*≥3). Bars represent the sem for each data point. Significant differences between untreated controls and treated samples are indicated (one-way ANOVA test; *P<0.05; **P<0.01; ***P<0.001).

To elucidate whether UBA1 could be exploited as a potential therapeutic target against the orthoflaviviruses, we examined the antiviral properties of two different small molecule compounds targeting this protein (Fig. 7). Both TAK-243 and PYR-41 led to significant decreases in virus yields in Vero cells (Fig. 7a–f). Nonetheless, TAK-243 exhibited larger efficacy than PYR-41, with significant differences observed between treated and untreated samples at most concentrations tested. Treatment with PYR-41 resulted in 7- and 32-fold decreases in ZIKV and USUV titres at 24 h post-infection, with IC_50_ values of 17 and 3.5 µM and selectivity indexes (SIs) of 4.1 and 20, respectively. Treatment with TAK-243 resulted in 47- and 900-fold decreases in ZIKV and USUV titres, with IC_50_ values of 161 and 45 nM and SI values of 5.4 and 19, respectively (Fig. S6). To assess whether TAK-243 also exhibited an antiviral activity during infection in a human cell context, we repeated this assay in A549 cells (Fig. 7g–i). We found similar results to those obtained above with IC_50_ values of 87 and 10 nM and SI values of 14 and 122 (Fig. S6). We also investigated the effect of these compounds at later time points (48 h post-infection), and similar results were observed in both A549 (Fig. 7j–l) and Vero cells (Fig. S7). These results suggest that UBA1 is a proviral factor for the orthoflaviviruses, further supporting the use of specific drugs as a conceivable therapeutic approach.

## Discussion

Here, we describe the interaction of host protein UBA1, a key enzyme in protein ubiquitylation, with ZIKV NS5 by co-IP and immunocytochemistry approaches (Figs1  2). We also demonstrate that UBA1 is a ZIKV proviral factor, as its overexpression leads to increases in virus titres and protein levels, and its knockdown negatively affects viral replication (Figs5  6). The analysis of infected cells by IFA suggests that both proteins primarily interact in the nuclear region, although cytoplasmic colocalization was observed in some cells (Fig. 3). While our data suggest that UBA1 is critical to the virus life cycle, further examination will be required to fully understand how its interaction with NS5 affects the host cell during infection. UBA1 nuclear activities are key to different cellular processes. Notably, histone ubiquitylation is required for cell cycle progression, with the inhibition of UBA1 causing cell cycle defects and replicative stress [21,22]. It has been shown that ZIKV infection alters cell cycle progression at multiple stages and that the expression of recombinant ZIKV NS5 alone also leads to an increase in the time needed to complete mitosis [33]. UBA1 has also been related to DNA damage repair mediated by ubiquitylation (dsDNA breaks, nt excision repair, etc.) supporting a major role in DNA stability and transcription [22,34]. Conversely, ZIKV infection induces cellular DNA damage, impairing cellular genome replication in human neuronal progenitor cells [35]. It remains to be elucidated, however, whether ZIKV interfering with cell cycle progression and host DNA repair machinery are related to NS5 interfering with UBA1.

Orthoflavivirus replication factories are located in convoluted ER-derived membranes in the cytosol of the infected cells. However, ZIKV NS5 is almost exclusively detected in the nucleus of an infected cell, compromising the identification of replication sites by immunofluorescence. By using an antibody recognizing dsRNA, we found that UBA1 colocalizes with or accumulates in close proximity to replicating viral RNA (Fig. 4). Previous studies have documented that other components of the UPS may contribute to the formation of the replication factories. Specifically, in addition to a key role in viral genome uncoating, VCP/p97 contributes to ER membrane rearrangements required for orthoflaviviral genome replication [36,39]. VCP is a highly conserved ATPase that acts as a chaperone, unfolding ubiquitinated proteins to facilitate their degradation in the proteasome [40]. In some orthoflaviviruses, VCP is recruited to replication factories to induce stress granule disassembly and promote viral RNA synthesis and translation. In this process, ubiquitinated proteins are accumulated adjacent to the viral replication organelles [41]. Altogether, these results support that protein ubiquitylation participates in the development of viral RNA replication sites.

One of the most relevant activities displayed by orthoflavivirus NS5s in the infected cell is blocking the IFN-1 signal transduction and therefore contributing to immune evasion [42]. In ZIKV and DENV, IFN-1 transduction is inhibited by the specific binding of NS5 to STAT2, mediating its subsequent proteasomal degradation [32]. In DENV, NS5 bridges the E3 ubiquitin-ligase enzyme UBR4 and STAT2, resulting in STAT2 degradation by the proteasome [43]. However, to our knowledge, there have not been yet identified any ubiquitinating enzymes contributing to STAT2 degradation during ZIKV infection. Preliminary data suggest that UBA1 inhibition in cells expressing NS5 results in a >4-fold decrease in STAT2 degradation (Fig. S8). Nevertheless, additional studies are needed to fully demonstrate that both UBA1 and STAT2 are physically connected by NS5 and whether this putative interaction is linked to degradation.

During ZIKV infection, UBA1 protein levels increase at least by twofold at 24 h post-infection. This increase may be related to the general activation of stress responses to the infection [44,45]. Alternatively, ZIKV may be specifically triggering UBA1 overexpression to promote its replication, as it can be inferred from overexpression and downregulation experiments. Viral titres were higher in cells transfected with pUBA1 and pFLAG-UBA1 constructs and lower in UBA1-depleted cells with DsiRNA molecules (Figs5  6). Despite exploiting the UPS pathway is a common strategy shared by many viruses to complete their life cycles [46], and UBA1 playing a critical role in such pathway, there is limited information on the role of UBA1 during infection by different viruses. Recent studies have established a possible connection between infections by severe acute respiratory syndrome coronavirus 2 (SARS-CoV-2) and Epstein–Barr virus (EBV) with UBA1 [47,48]. In both cases, it has been reported that patients infected with these viruses develop symptoms resembling the vacuoles, E1 enzyme, X-linked, autoinflammatory and somatic (VEXAS) syndrome, a recently identified autoinflammatory disease, which is generally caused by deficiencies in UBA1 expression [49]. The most common polymorphism in VEXAS affects the second translational initiation codon. Hence, the transcriptional variant UBA1b is lost, and a new isoform (UBA1c) is detected in these patients [50]. Altogether, these evidences support that SARS-CoV-2 and EBV may be interfering with the correct function of UBA1 to promote their survival.

A prospective therapeutic approach may entail the development of drugs directly targeting UBA1 or disrupting its interaction with NS5. Two UBA1-targeting drugs have been used in this study, TAK-243 and PYR-41. While PYR-41 exerts a modest antiviral activity, TAK-243 elicits a robust antiviral behaviour against both ZIKV and USUV (Fig. 7). A possible explanation for such a difference in their efficacy might be related to their mechanisms of action. PYR-41 is a nitropyrazone-based inhibitor, which is irreversibly bound to the catalytic cysteine in UBA1 [21]. Conversely, TAK-243 is an adenosine sulfamate inhibitor, which specifically targets the adenylate-binding site in UBA1. As a result, TAK-243 is covalently bound to the C-terminus of ubiquitin, forming a TAK-243-ubiquitin adduct, which blocks the activation of E1 enzymes [51]. A tantalizing possibility is that TAK-243 might be triggering different conformational changes in UBA1 than PYR-41 or masking interacting epitopes and hence impairing NS5 binding. Since PYR-41 covalently alters UBA1, while TAK-243 chemically binds to ubiquitin, another possibility is that free ubiquitin, even in the absence of a functional UBA1 molecule, is critical to virus replication. Owing to the fact that it has been advanced to the clinical treatment of different types of cancers (phase 1), including solid tumours and various types of blood cancer, TAK-243 is a promising drug that could be explored as a potential anti-orthoflavivirus drug [21]. Since aberrant protein ubiquitylation is typically found in the cancer cell and the E1-activating step is almost exclusively catalysed by UBA1, novel small molecule compounds are continuously developed against this protein, further expanding future therapeutic possibilities [21,52].

A hindrance to the use of UBA1-targeting drugs is potential undesirable side effects. Alterations in the protein levels and function of UBA1 have been related to multiple neurodegenerative disorders, such as spinal muscular dystrophy and Alzheimer’s disease [21,22]. Lower levels of endogenous UBA1 in the brain also correlate with increased levels of mutant huntingtin protein in a mouse model of Huntington’s disease [53]. Thus, it is conceivable that UBA1 inhibitors may exacerbate the potential neurological damage in the context of a neurotropic viral infection. Alternative therapeutic strategies could involve molecules specifically disrupting the NS5–UBA1 interaction without affecting UBA1 activity. Drugs against other enzymes participating in ubiquitylation or degradation by the proteasome (e.g. VCP and deubiquitinases) could be studied as alternative host targets for antivirals [25].

In summary, here, we demonstrate that orthoflaviviral NS5 interacts with UBA1, a key factor in protein ubiquitylation, and this interaction primarily takes place in the nucleus of the infected cell. Overexpression and RNAi-mediated downregulation experiments suggest that UBA1 plays a proviral role during ZIKV infection, supporting the development of antiviral therapies aiming at disrupting this virus–host interaction.

## supplementary material

10.1099/jgv.0.002063 Supplementary Material 1.

## Abbreviations

co-IP: co-immunoprecipitation
DENV: dengue virus
DMEM: Dulbecco’s modified Eagle’s medium
1× DPBS: 1× Dulbecco’s PBS
DsiRNA: Dicer-substrate small interfering RNA
ER: endoplasmic reticulum
EYFP: enhanced yellow fluorescent protein
IC_50_: 50% inhibitory concentration
IDT: Integrated DNA Technologies
IFA: immunofluorescence assay
MTase: methyltransferase
NS5: non-structural protein 5
PBS-T: PBS containing 0.0% Tween-20
RdRp: RNA-dependent RNA polymerase
RNAi: RNA interference
SI: selectivity index
UBA1: ubiquitin-like modifier-activating enzyme 1
UPS: ubiquitin-proteasome system
USUV: Usutu virus
VCP: valosin-containing protein
VEXAS: vacuoles, E1 enzyme, X-linked, autoinflammatory and somatic
WB: Western blot
WNV: West Nile virus
ZIKV: Zika virus
