# Supplementary material for: Ubiquitin-like modifier-activating enzyme 1 interacts with Zika virus NS5 and promotes viral replication in the infected cell
Source: J Gen Virol. 2025 Jan 8;106(1):002063. doi: 10.1099/jgv.0.002063 (PMC11708914; doi:10.1099/jgv.0.002063)

# Supplementary information

## Ubiquitin-like modifier activating enzyme 1 interacts with Zika virus NS5 and promotes viral replication in the infected cell

Imanol Rodrigo[1,2], Laura Albentosa-González[1,2,3], María José Romero de Ávila[1,2], Maria Rosaria Bassi[4], Raquel Navarro Sempere[5], Pilar Clemente-Casares[1,2,3,#], Armando Arias[1,2,6,#]

# Address correspondence to:

Armando Arias, armando.arias@uclm.es; Pilar Clemente-Casares, pilar.ccasares@uclm.es

## SUPPLEMENTARY FIGURE LEGENDS

**Fig. S1.** Identification of UBA1 as an interacting factor of ZIKV RdRp domain. To this study, we prepared plasmids encoding the RdRp domain of ZIKV NS5 (residues 276 to 903). With this aim, we designed forward (5'-GCCGGAATTCGCCACC**ATG**AAGATCATTGGTAACCGCATTGAAAG-3', bold-highlighted the initiation codon preceding the RdRp sequence) and reverse primers (5'-CAGCACTCCAGGTGTAGACCCTTC-3') that were used to amplify the RdRp-coding region. For amplification, viral RNA was reverse transcribed using Superscript III (Invitrogen) and then amplified by PCR using AccuPrime Taq DNA Polymerase, high fidelity (Invitrogen), following protocols provided by the manufacturer. Amplicons containing 3' adenine overhangs were cloned using TA cloning into the pcDNA6.2/C-EmGFPGW/TOPO vector from the Vivid Colors kit (Invitrogen), following the specific instructions for this reagent. We obtained constructs expressing an N-terminal RdRp fused to a C-terminal GFP molecule that were used for subsequent assays. (a) Identification of proteins specifically binding ZIKV RdRp in a co-immunoprecipitation assay followed by mass spec. Coimmunoprecipitation (co-IP) assays were performed by using GFP-Trap Magnetic Particles (Chromotek), and interacting factors were eluted in 200 mM glycine pH 2.5, and neutralised with 1 M Tris pH 10.4. Host factors enriched at least 4-fold in co-IPs from cells expressing RdRp-GFP relative to cells expressing GFP. Co-IP samples were mass spec analysed by the DTU Core Proteomics service and the ratios of identified proteins were given. (b) UBA1 band intensity is 4.1-fold larger in co-IP of cells transfected with a plasmid encoding RdRp-GFP than in cells transfected with GFP alone.

**Fig. S2. UBA1 specifically interacts with ZIKV NS5.** (a) UBA1 is specifically co-immunoprecipitated with HA-tagged ZIKV NS5 but not LacZ. 293T cells were transfected with constructs expressing HA-tagged ZIKV NS5, EYFP (pEYFP-N1, shown as pEYFP), an empty vector (pcDNA3.1, shown as pcDNA) or HA-tagged LacZ (pCruz HA-LacZ, Santa Cruz Biotechnologies). HA-tagged proteins were immunoprecipitated with anti-HA agarose beads.

(b) UBA1 coprecipitated with recombinant His-tagged ZIKV NS5 (His-ZK NS5) in assays using Ni-NTA Agarose resin (Invitrogen). To the assay, 3 mg of whole cell protein, extracted from 293T cells, were incubated in the presence of either 90 μg of His-tagged dihydrofolate reductase (His-DHFR), His-ZIKV NS5 or untagged recombinant bovine serum albumin (BSA). To the expression and purification of His-DHFR, we used the plasmid pDHFR in E. coli BL21, following the instructions provided by the manufacturer (Bio-Rad).

**Fig. S3. Recombinant ZIKV NS5 colocalises with UBA1 in transfected cells.** Fluorescence emitted by UBA1 and HA-tagged NS5 detection overlap in the nuclear region of HEK-293T cells. A close-up view of colocalisation in each cell is provided. A total of 1.5 x $10^5$ HEK 293T cells were seeded on a coverslip. At 24 h, 1 μg of a plasmid encoding HA-tagged ZIKV NS5 were transfected, using lipofectamine 3000 and protocols suggested by the manufacturer (Invitrogen). IFA to the detection of ZIKV NS5 and UBA1 with specific mouse anti-UBA1 and rabbit anti-NS5 antibodies.

**Fig. S4. Toxicity and silencing efficacy of DsiUBA1 in cell culture.** (a) Number of metabolically active cells after transfection with a specific (UBA1) or an unspecific (NegC) DsiRNA. 2 x $10^4$ cells were doubly transfected, leaving a 24-h interval between transfections, with either 1.5 (3 in total) or 5 (10 in total) pmol of each DsiRNA applied to each well. At 48 hours, the relative amount of viable cells is calculated by using the *CellTiter-Blue Cell Viability Assay kit* as suggested by the manufacturer. Values are represented as a percentage relative to mock-treated cells. (b) UBA1 detection in cells by immunofluorescence assay at 24 or 48 h postransfection. in MocK-transfected cells or cells treated with either a specific (DsiUBA1) or a non-human target (DsiNegC) DsiRNA. To the detection of UBA1 (red), a specific mouse anti-UBA1 antibody was used. Nuclei were stained with DAPI (blue).

**Fig. S5. Enhanced ZIKV NS5 protein levels in cells overexpressing UBA1.** ZIKV NS5

protein levels in cells previously transfected with pUBA1 or an empty vector (pcDNA). (a) WB using an anti-ZIKV NS5 antibody; as a loading reference control an anti-tubulin antibody was used. (b) Graph illustrating NS5 protein levels relative to those detected in Mock-transfected cells (empty) at 24 h post-infection. GAPDH is used as a loading control.

**Fig. S6. Determination of the 50% cytotoxicity concentration ($CC_{50}$) and 50% inhibitory concentration ($IC_{50}$) values for UBA1-targeting drugs.** The 50% cytotoxicity concentration ($CC_{50}$) and the 50% inhibitory concentration ($IC_{50}$) values for each drug and virus are given. (a, d, g) Relative number of metabolically live Vero (a, d) or A549 (g) cells after treatment during 24 h with increasing concentrations of PYR-41 (a) and TAK-243 (d, g). The relative amount of metabolically active cells in treated samples is calculated by using the *CellTiter-Blue Cell Viability Assay* kit as suggested by the manufacturer (Promega). Values are represented as a percentage relative to mock-treated cells. The $CC_{50}$ values for each drug and cell line are provided in the graphs. (b, c, e, f, h, i) $IC_{50}$ values for PYR-41 (b, c) and TAK-243 (e, f, h, i) against ZIKV and USUV in cell culture. The antiviral activity was examined by plotting the virus titre ($TCID_{50}$) values obtained at increasing concentrations of drugs. The $IC_{50}$ values obtained are provided in the corresponding graphs. (b, c) Virus titres in PYR-41-treated Vero cells, previously infected with ZIKV (b) or USUV (c). (e, f) Virus titres TAK-243-treated Vero cells, previously infected with ZIKV (e) or USUV (f). (h, i) Virus titres in TAK-243-treated A549 cells, previously infected with ZIKV (h) or USUV (i). Each value in the graphs is the average of at least three independent biological replicas ($n \geq 3$). Standard error of the mean (SEM) values are represented as bars for each data point.

**Fig. S7. UBA1 drugs PYR-41 and TAK-243 inhibit ZIKV and USUV replication in Vero cells at 48 h post-infection.** (a, d) Relative number of metabolically live Vero cells after treatment with increasing concentrations of PYR-41 (a) and TAK-243 (d) hours. The relative amount of metabolically active cells after 48 h in the presence of increasing concentrations of each drug is calculated by using the *CellTiter-Blue Cell Viability Assay* kit as suggested by the

manufacturer (Promega). Values are represented as a percentage relative to mock-treated cells. (b, c, e, f) The antiviral activity was examined by 50% tissue culture infectious dose (TCID50) assays of samples collected from infected cells treated with increasing concentrations of drugs. To this, Vero cells were inoculated with either ZIKV at a multiplicity of infection (MOI) of 0.2 $TCID_{50}$/cell or USUV at 0.02 $TCID_{50}$/cell. At 48 h post infection, the cellular supernatants were collected and the virus titres quantified. (b, c) Virus titres at 48 h post-infection in PYR-41-treated cells, previously infected with ZIKV (b) or USUV (c). (e, f) Virus titres at 48 h post-infection in TAK-243-treated cells, previously infected with ZIKV (e) or USUV (f). Each value in the graph is the average of at least three independent biological replicas ($n \geq 3$). Bars are representing the standard error of the mean (SEM) for each data point. Significant differences between untreated controls and treated samples are indicated (One-way ANOVA test; *, $p < 0.05$; **, $p < 0.01$; ***, $p < 0.001$).

**Fig. S8. TAK-243 leads to a modest increase of STAT2 in NS5-expressing cells.** WB of STAT2 at 48 h post-transfection in cells either expressing recombinant HA-tagged LacZ or ZIKV NS5. At 6 h post-transfection, cell supernatants containing the transfection complexes were removed and 4 ml of fresh media containing no drug (negative symbols, −) or 300 nM TAK-243 (positive symbols, +) were added. As a loading control an anti-GAPDH antibody was used.

# Figure S1

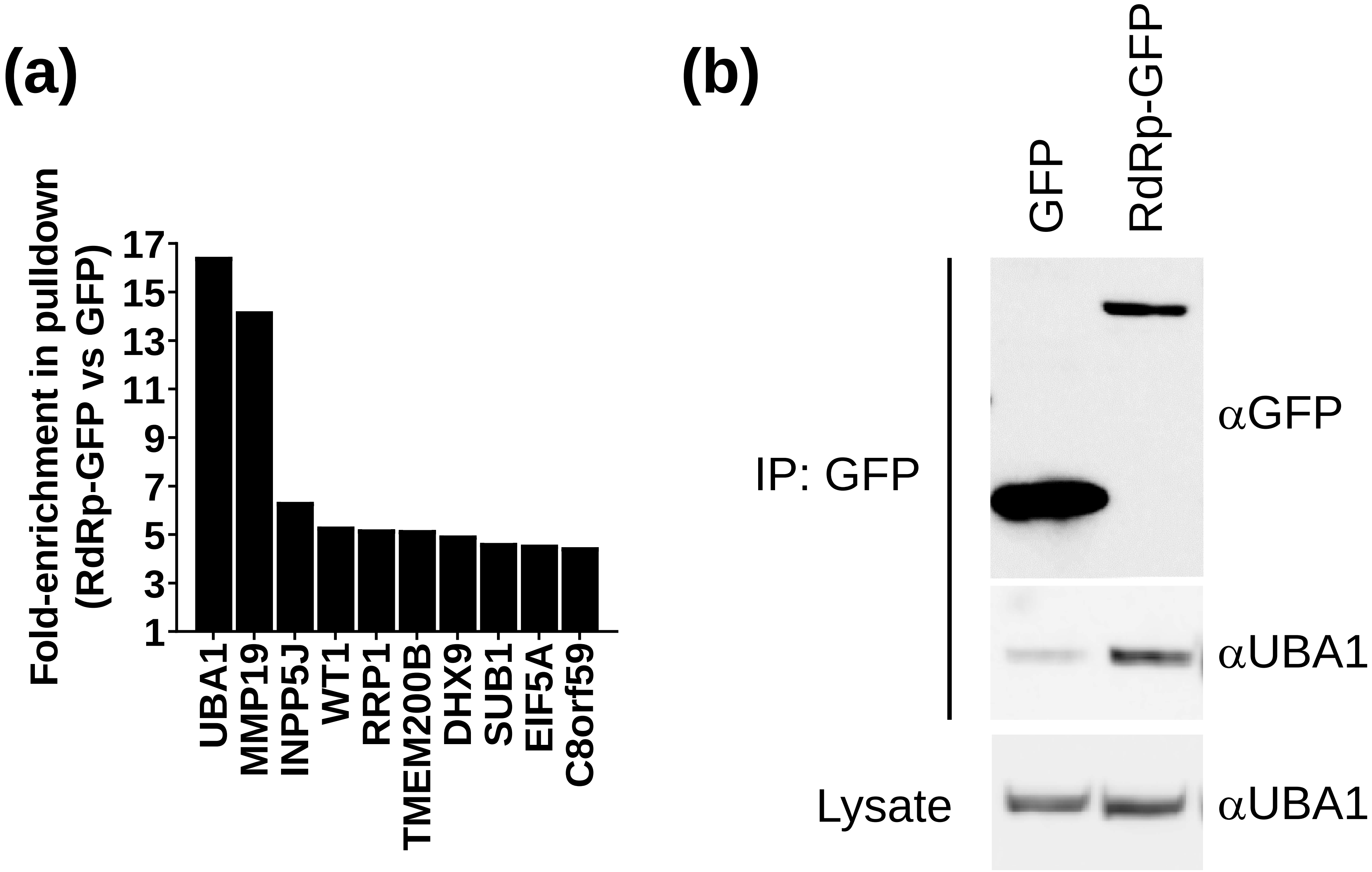

(a)
Fold-enrichment in pulldown (RdRp-GFP vs GFP)
1
3
5
7
9
11
13
15
17
UBA1
MMP19
INPP5J
WT1
RRP1
TMEM200B
DHX9
SUB1
EIF5A
C8orf59
(b)
GFP
RdRp-GFP
IP: GFP
Lysate
αGFP
αUBA1
αUBA1

# Figure S2

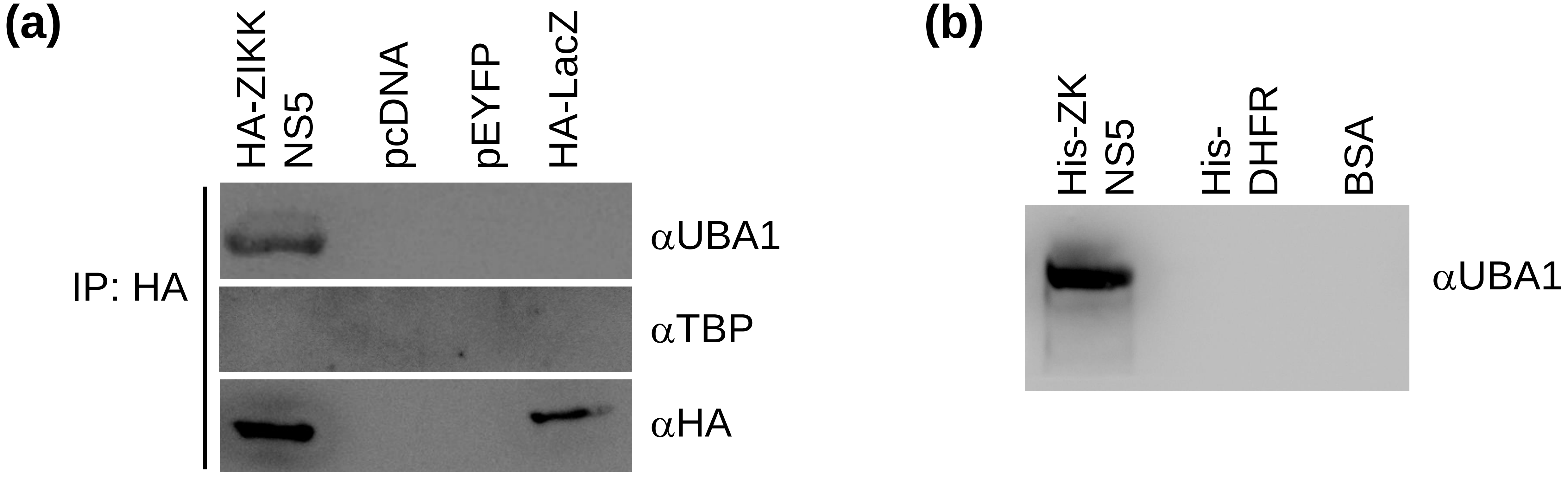
(a)
HA-ZIKK NS5
pcDNA
pEYFP
HA-LacZ
IP: HA
αUBA1
αTBP
αHA
(b)
His-ZK NS5
His-DHFR
BSA
αUBA1

# Figure S3

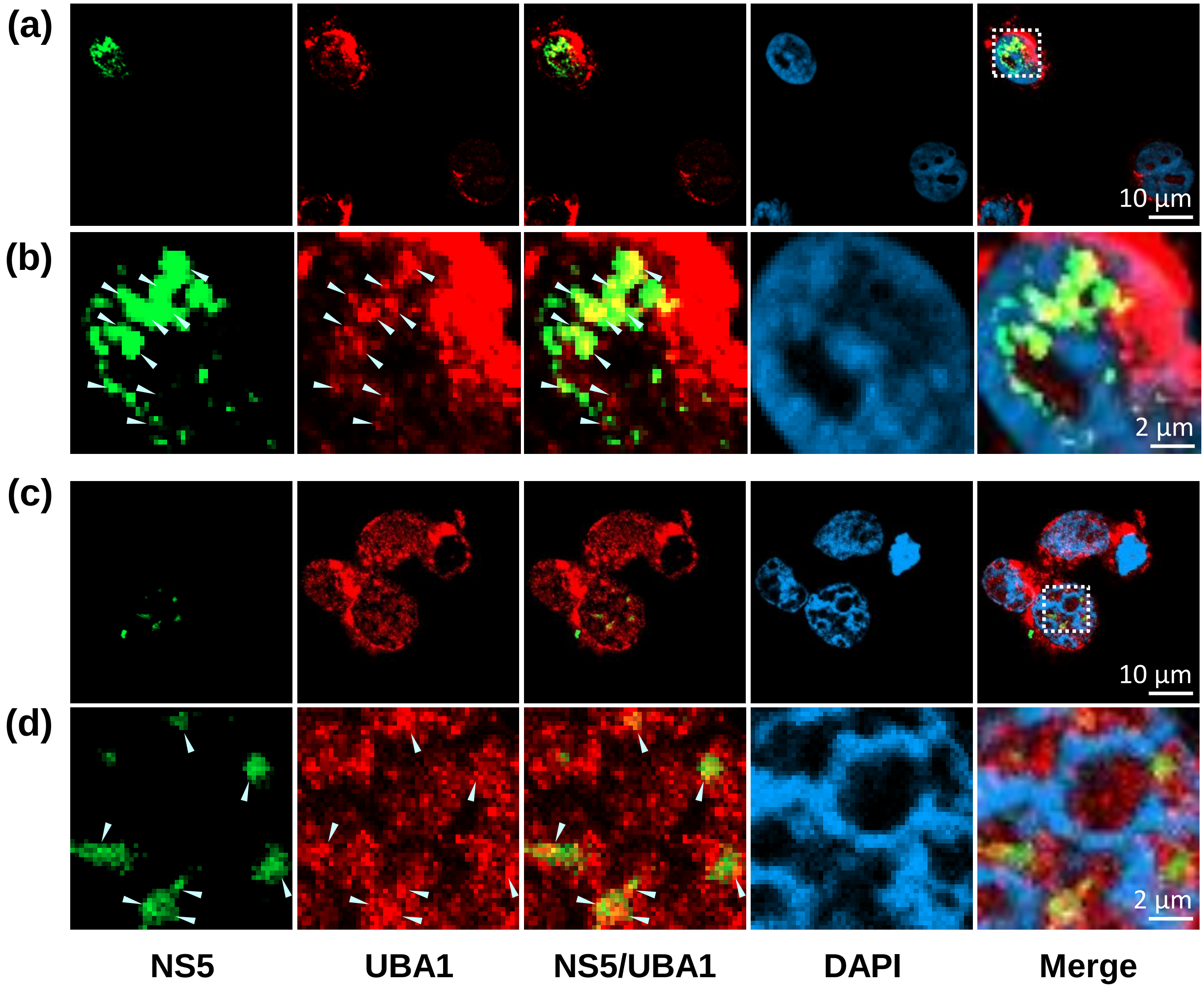

# Figure S4

(a)

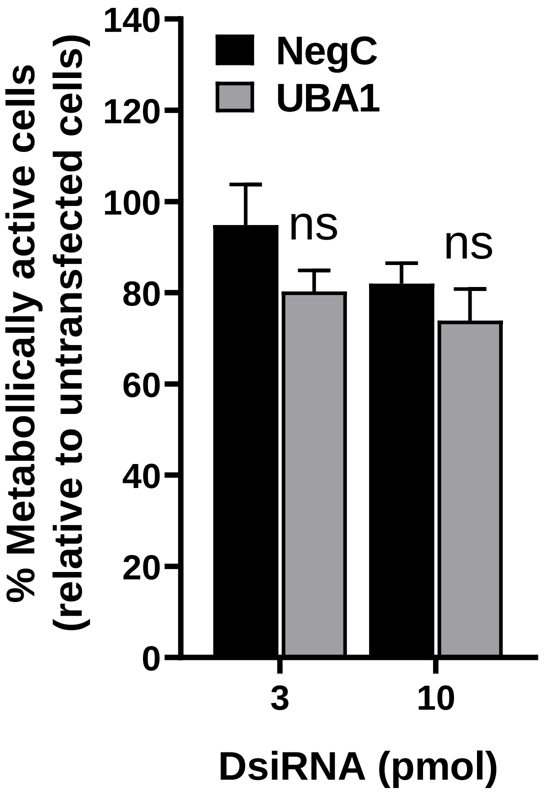

140
120
100
80
60
40
20
0
% Metabolically active cells
(relative to untransfected cells)
NegC
UBA1
ns
ns
3
10
DsiRNA (pmol)


(b)

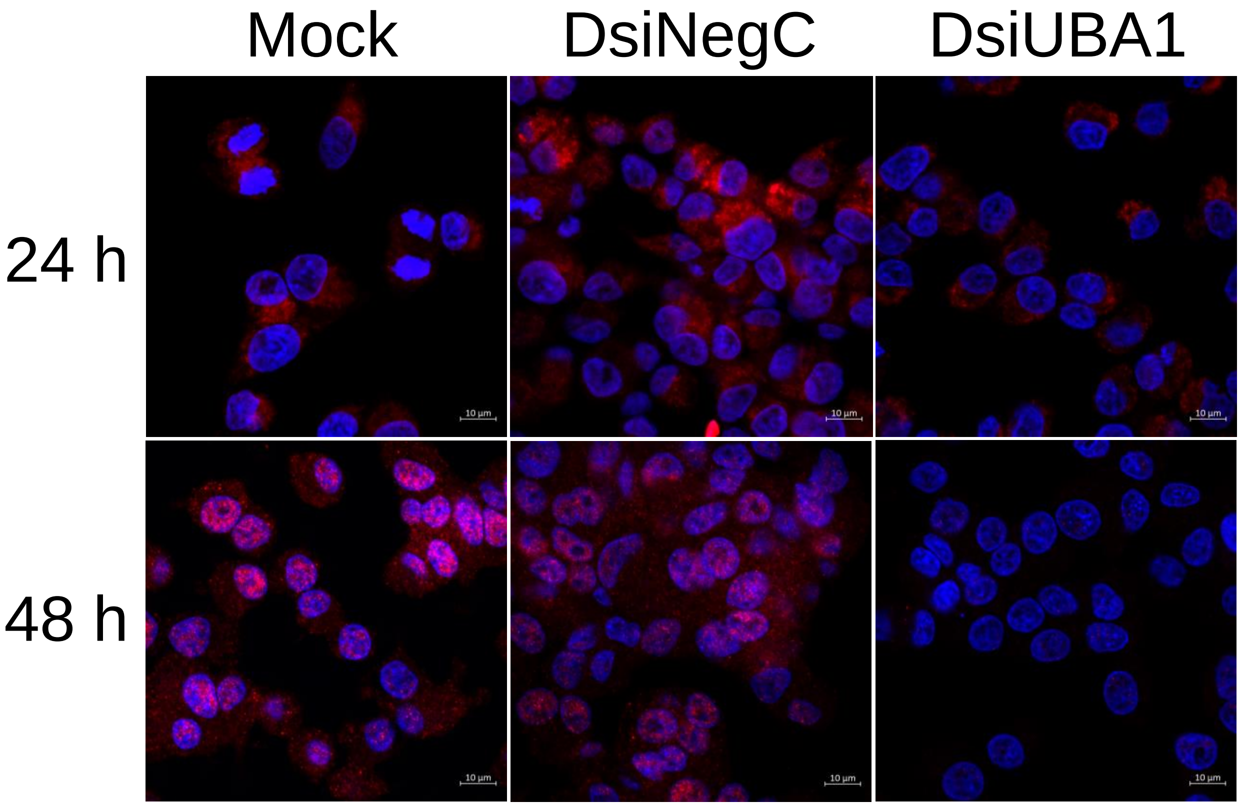

Mock
DsiNegC
DsiUBA1
24 h
48 h

# Figure S5

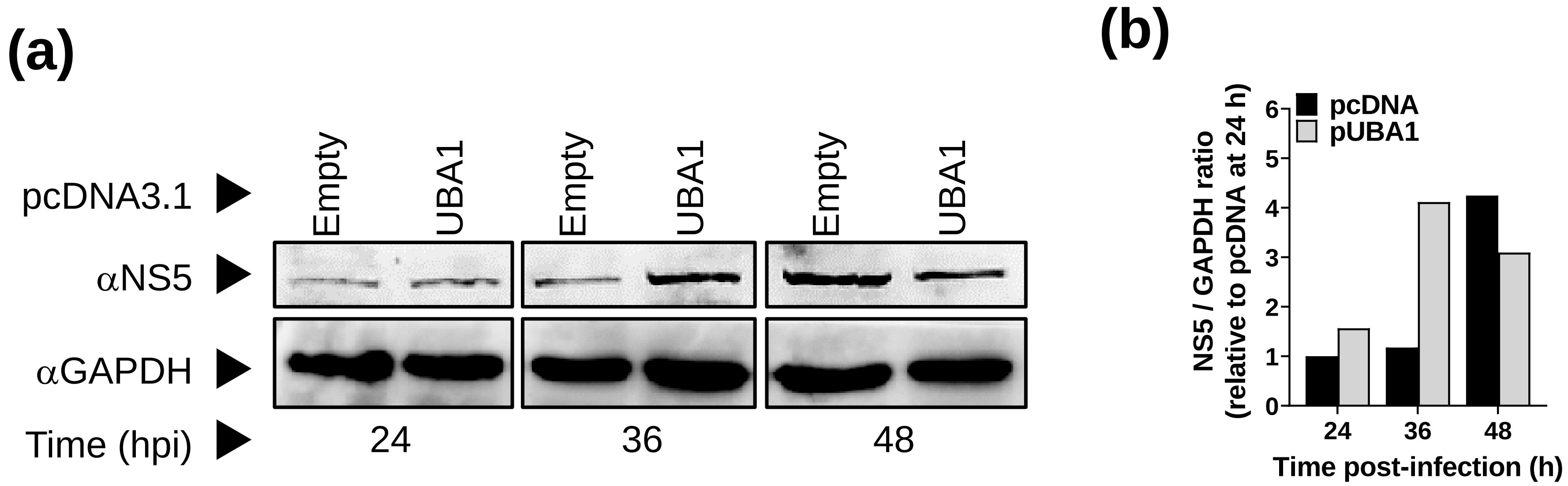
(a)
pcDNA3.1
Empty
UBA1
Empty
UBA1
Empty
UBA1
αNS5
αGAPDH
Time (hpi)
24
36
48
(b)
pcDNA
pUBA1
NS5 / GAPDH ratio
(relative to pcDNA at 24 h)
0
1
2
3
4
5
6
24
36
48
Time post-infection (h)

# Figure S6

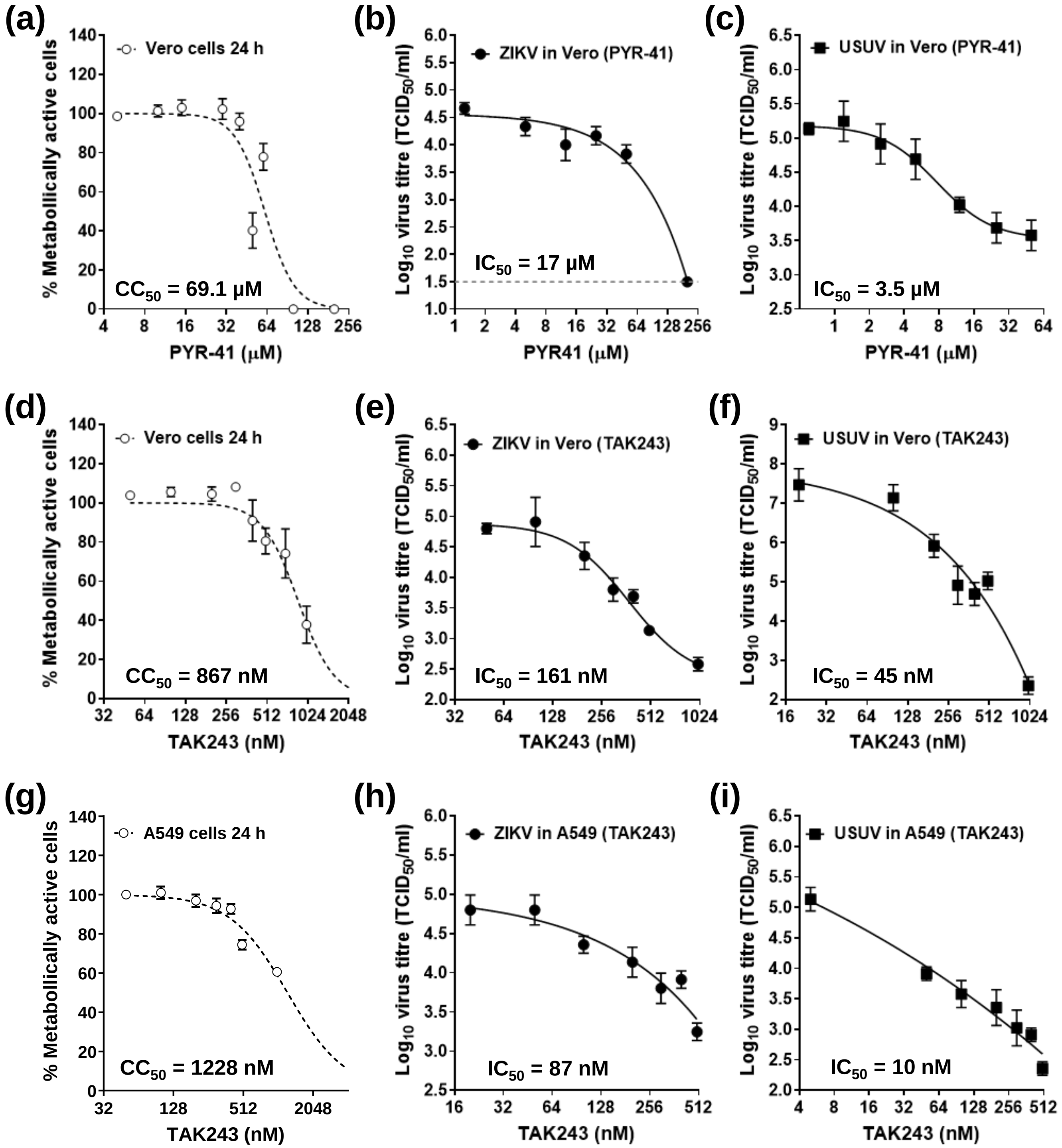

(a)
Vero cells 24 h
% Metabollically active cells
CC50 = 69.1 µM
PYR-41 (µM)
(b)
ZIKV in Vero (PYR-41)
Log10 virus titre (TCID50/ml)
IC50 = 17 µM
PYR41 (µM)
(c)
USUV in Vero (PYR-41)
Log10 virus titre (TCID50/ml)
IC50 = 3.5 µM
PYR-41 (µM)
(d)
Vero cells 24 h
% Metabollically active cells
CC50 = 867 nM
TAK243 (nM)
(e)
ZIKV in Vero (TAK243)
Log10 virus titre (TCID50/ml)
IC50 = 161 nM
TAK243 (nM)
(f)
USUV in Vero (TAK243)
Log10 virus titre (TCID50/ml)
IC50 = 45 nM
TAK243 (nM)
(g)
A549 cells 24 h
% Metabollically active cells
CC50 = 1228 nM
TAK243 (nM)
(h)
ZIKV in A549 (TAK243)
Log10 virus titre (TCID50/ml)
IC50 = 87 nM
TAK243 (nM)
(i)
USUV in A549 (TAK243)
Log10 virus titre (TCID50/ml)
IC50 = 10 nM
TAK243 (nM)

# Figure S7

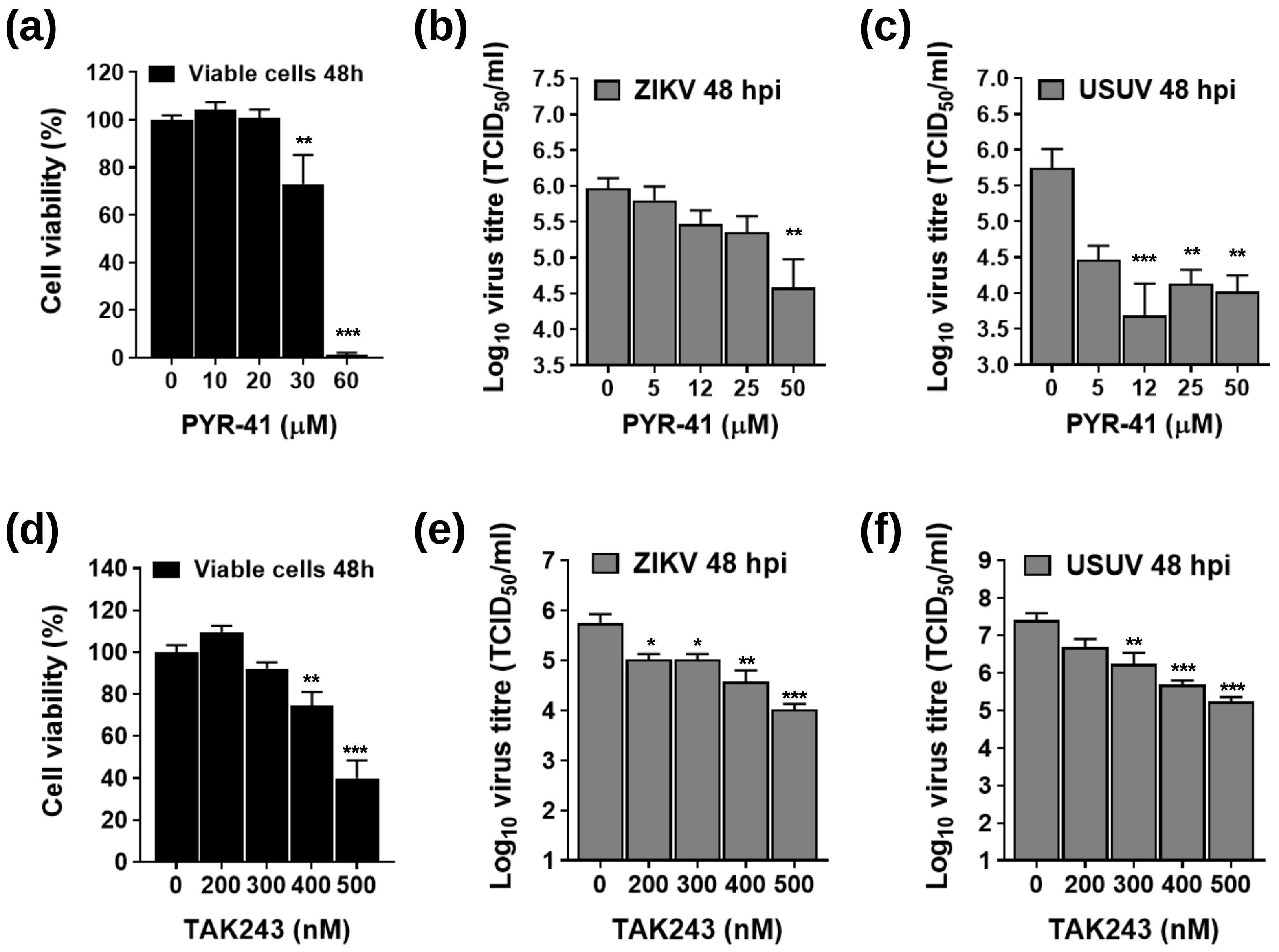

(a)
Viable cells 48h
Cell viability (%)
120
100
80
60
40
20
0
**
***
0 10 20 30 60
PYR-41 (μM)
(b)
ZIKV 48 hpi
Log10 virus titre (TCID50/ml)
7.5
7.0
6.5
6.0
5.5
5.0
4.5
4.0
3.5
**
0 5 12 25 50
PYR-41 (μM)
(c)
USUV 48 hpi
Log10 virus titre (TCID50/ml)
7.0
6.5
6.0
5.5
5.0
4.5
4.0
3.5
3.0
*** ** **
0 5 12 25 50
PYR-41 (μM)
(d)
Viable cells 48h
Cell viability (%)
140
120
100
80
60
40
20
0
**
***
0 200 300 400 500
TAK243 (nM)
(e)
ZIKV 48 hpi
Log10 virus titre (TCID50/ml)
7
6
5
4
3
2
1
* * ** ***
0 200 300 400 500
TAK243 (nM)
(f)
USUV 48 hpi
Log10 virus titre (TCID50/ml)
9
8
7
6
5
4
3
2
1
** *** ***
0 200 300 400 500
TAK243 (nM)

# Figure S8

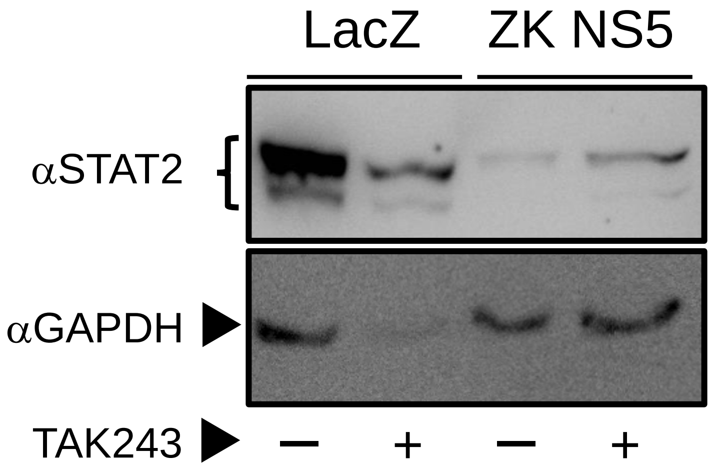

Supplement: Supplementary Material 1. [file jgv-106-02063-s001.pdf]
